# Effect of workplace physical activity interventions on the cardio-metabolic health of working adults: systematic review and meta-analysis

**DOI:** 10.1186/s12966-019-0896-0

**Published:** 2019-12-19

**Authors:** Rubina Mulchandani, Ambalam M. Chandrasekaran, Roopa Shivashankar, Dimple Kondal, Anurag Agrawal, Jeemon Panniyammakal, Nikhil Tandon, Dorairaj Prabhakaran, Meenakshi Sharma, Shifalika Goenka

**Affiliations:** 1 Indian Institute of Public Health-Delhi, Public Health Foundation of India, Gurgaon, India; 2 Centre for Chronic Disease Control, New Delhi, India; 3 CSIR Institute of Genomics and Integrative Biology, New Delhi, India; 4 Sree Chitra Tirunal Institute for Medical Sciences and Technology, Government Medical College, Thiruvananthapuram, Kerala India; 5 Public Health Foundation of India, Gurgaon, India; 6 All India Institute of Medical Sciences, Delhi, India; 7 Indian Council of Medical Research, New Delhi, India

**Keywords:** Physical activity, Worksite interventions, Cardiovascular disease

## Abstract

**Background:**

Adults in urban areas spend almost 77% of their waking time being inactive at workplaces, which leaves little time for physical activity. The aim of this systematic review and meta-analysis was to synthesize evidence for the effect of workplace physical activity interventions on the cardio-metabolic health markers (body weight, waist circumference, body mass index (BMI), blood pressure, lipids and blood glucose) among working adults.

**Methods:**

All experimental studies up to March 2018, reporting cardio-metabolic worksite intervention outcomes among adult employees were identified from PUBMED, EMBASE, COCHRANE CENTRAL, CINAHL and PsycINFO. The Cochrane Risk of Bias tool was used to assess bias in studies. All studies were assessed qualitatively and meta-analysis was done where possible. Forest plots were generated for pooled estimates of each study outcome.

**Results:**

A total of 33 studies met the eligibility criteria and 24 were included in the meta-analysis. Multi-component workplace interventions significantly reduced body weight (16 studies; mean diff: − 2.61 kg, 95% CI: − 3.89 to − 1.33) BMI (19 studies, mean diff: − 0.42 kg/m^2^, 95% CI: − 0.69 to − 0.15) and waist circumference (13 studies; mean diff: − 1.92 cm, 95% CI: − 3.25 to − 0.60). Reduction in blood pressure, lipids and blood glucose was not statistically significant.

**Conclusions:**

Workplace interventions significantly reduced body weight, BMI and waist circumference. Non-significant results for biochemical markers could be due to them being secondary outcomes in most studies. Intervention acceptability and adherence, follow-up duration and exploring non-RCT designs are factors that need attention in future research.

Prospero registration number: CRD42018094436.

## Background

### Physical activity as a modifiable health behavior for cardiovascular disease (CVD) prevention

According to the INTERHEART study, physical inactivity is one of the 9 major modifiable risk factors responsible for CVDs in both sexes worldwide [1]. It is responsible for 10% of the premature mortality, 6% of coronary heart disease burden and 7% of the diabetes burden worldwide [2]. Approximately 3.2 million annual deaths are attributable to insufficient activity [3] and 25% reduction in inactivity can avert 1.3 million deaths annually [2]. Physical activity (PA) aids in better glycemic control and it is a vital component of diabetes prevention and management [4]. The World Health Organization (WHO) now recommends 150–300 min of moderate to vigorous aerobic physical activity (MVPA) for adults aged 18–64 [5]. Some of the most common reasons for inactivity among adults are an unsupportive social and physical environment [6, 7] and lack of time [8]. Adults in urban areas spend almost 77% of their waking time being inactive at work or otherwise, leaving little time for exercise [9, 10].

Worksite physical activity programs are specifically designed with the aim of enhancing employee physical activity levels and improving their dietary behavior at the workplace [11]. Worksite settings provide effective channels to reach defined populations, disseminate information, create an effective medium for program delivery and study the impact to maximize benefits [12, 13]. These can be suitable settings for advocating an active lifestyle, improving employee productivity and reducing healthcare costs [14, 15]. Contemporary workplaces are thus ideal for interventions that promote higher levels of physical activity amongst employees, to improve health and optimize performance [16].

### Rationale for the current systematic review and meta-analysis

A number of narrative and systematic reviews have demonstrated the positive effect of various worksite physical activity interventions on physical activity, productivity and cost outcomes [17–25]. However, only a handful of them have comprehensively evaluated the effects of these interventions on the major measurable cardiovascular disease markers. The last comprehensive review on the topic was done in 2010, included only randomized controlled trials (RCTs) and did not meta-analyze the effects [26]. Worksite PA interventions can provide an effective lever to address the CVD burden. However, the effectiveness of these interventions needs to be quantified. Given the availability of numerous primary studies in the area, it becomes imperative to present not only an overview but also obtain an overall quantitative estimate of intervention effects from different studies, both randomized and non-randomized.

Therefore, we aimed to undertake a comprehensive and systematic synthesis of literature and meta-analysis of available evidence, to obtain a holistic view, of the potential of worksite PA interventions in improving the cardio-metabolic health of working adults.

### Objective

To summarize evidence for the effect of worksite physical activity interventions on CVD risk markers (body weight, waist circumference, body mass index, blood pressure, lipids and blood glucose) among working adults and describe the intervention approaches used in the different studies.

### Research question

Do worksite physical activity interventions lower the cardio-metabolic disease risk of adults?

## Methods

The review methodology was registered with PROSPERO (registration ID: CRD42018094436) and has been described in detail in the protocol [27].

### Search strategy and inclusion of studies

We searched Cochrane Central, PUBMED, CINAHL, PSYCINFO and EMBASE to identify relevant studies on workplace physical activity interventions published till March 2018 using keywords like “workplace”, “workers”, “physical activity”, “exercise”, “wellness”, “counseling”, “RCTs”, “trials” etc. A comprehensive strategy was prepared by one researcher (RM) and reviewed by the second (CS) researcher. The PUBMED search strategy is illustrated in the Additional File 1. It was then modified as per the indexing system of other databases.

Eligibility criteria for inclusion of studies

- Study designs- Experimental study designs with a comparator group including randomized controlled trials, controlled trials, cluster RCTs, quasi-experimental studies; a comparator could be no intervention, minimal intervention, usual care, waitlisted control.
- Study populations- Studies involving individuals aged 18 and above; healthy populations as well as populations at risk of CVD were included
- Study outcomes- Studies reporting any of the CVD outcomes (body weight, body fat, waist circumference, BMI, blood pressure, plasma glucose, lipids and triglycerides)
- Study interventions- Workplace studies implementing physical activity based interventions targeting inactivity to improve the cardio-metabolic disease markers (anthropometric and biochemical) in adult employees

Exclusion criteria: Studies not published in the English language, those with a follow-up period of less than 6 months, observational studies and experimental studies without a comparator.

Referencing software Zotero was used to import the search results and remove the duplicates. Titles and abstracts of all the retrieved articles were screened independently by RM; CS independently screened 10% of the citations. The reference list of relevant studies obtained was further hand searched. Full texts of eligible studies were screened by RM and reviewed by CS. Wherever data for meta-analysis was unavailable in the public domain, the study authors were electronically contacted.

### Data extraction, quality assessment and analysis

Data extraction was performed independently by the two researchers. Disagreements were resolved within the team. Items in the data extraction form were prepared by RM using the Cochrane Handbook recommendations and were verified by CS. Outcomes were appropriately converted to the International System of Units for studies that reported them in other units. Findings from all the studies were included in the narrative synthesis. Review Manager (RevMan version 5.3) was used for the meta-analysis. The inverse-variance method was used to combine effect sizes using the random effects models (REMs) [28]. The treatment effect was reported as mean difference (MD) with 95% confidence intervals (CIs) wherein CIs excluding 0 were considered to be statistically significant. Forest plots were generated using RevMan to compare each of the proposed outcome measures in the intervention vs the control groups in the included studies. Studies that did not provide this data were excluded from the meta-analysis. REMs were used to report the overall mean difference with 95% CIs. The confidence intervals for each study in the meta-analysis were observed for their level of overlap, for a visual assessment of heterogeneity. I^2^ values, defined as ‘the percentage of variability in effect estimates that is due to heterogeneity rather than sampling error’, were used to determine the magnitude of variation beyond chance. It is calculated as [(Q-df)/Q]*100 where Q is the chi-square statistic and df is its degrees of freedom. A chi-square *p*-value of less than 0.05 was considered statistically significant for the presence of heterogeneity. Degree of heterogeneity was ascertained based on the cut-offs mentioned in the Cochrane handbook (0–40%: not important, 30–60%: moderate, 50–90%: substantial, 75–100%:considerable heterogeneity) [29].

The intervention effects on various CVD markers were also assessed under the sub-groups of study design (RCTs vs cRCTs), duration (6–12 months vs > 12 months), intervention type (predominantly educational vs predominantly behavioral change vs predominantly environmental changed based) and employee health status (all employees vs those at risk of CVD). The chi-square test p-value for sub-group differences was assessed for significant sub-group effects (a *p* < 0.05 indicates significant sub-group effect).

We classified the various intervention approaches used in the included studies based on a 2012 review by Heath et al. [30]. The interventions were broadly categorized as follows:

Campaigns and informational approaches: This involves information dissemination through different mediums like text messages, emails, newspapers, television, radio, to raise awareness and encourage a change in health behaviors mainly increasing activity and improving diet.

Behavioral and social approaches: This involves a change in individual behavior to incorporate more physical activity in their regular routine through goal setting, peer support and self-rewards. It can be implemented in groups (through technological means) as well as on an individual level with the help of a health provider/trainer and personalized activity plans.

Environmental and policy approaches: This involves making the office infrastructure and physical environment more activity friendly through construction of walking paths, changes to the vending machines, introduction of ergonomic workstations, break rooms, fitness facilities etc.

The Cochrane risk of bias tool [31] was used to assess the bias in included studies. The assessment was independently performed by RM and CS and disagreements were resolved by consensus. Possible publication bias among the studies was visually assessed using funnel plots.

## Results

### Literature search and characteristics of included studies

**Fig. 1:**
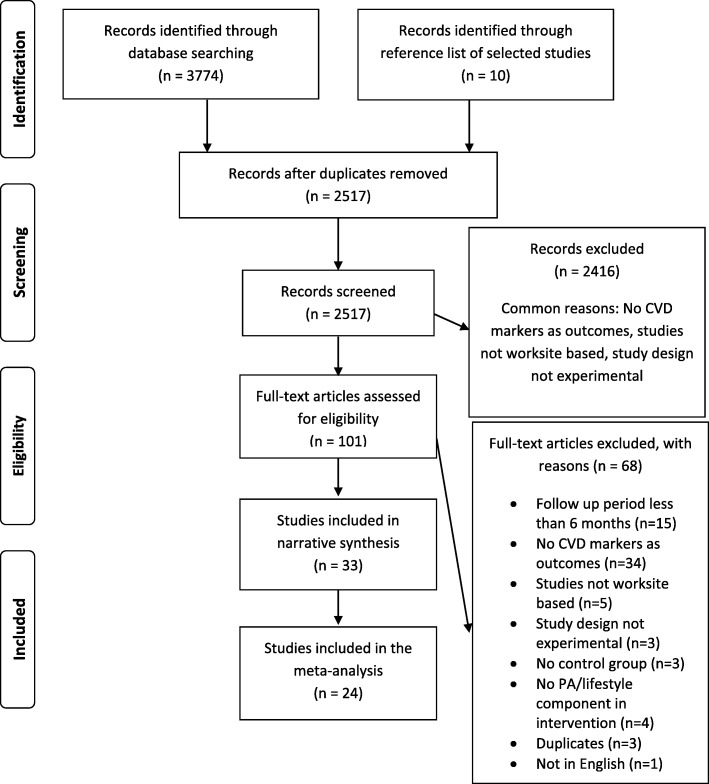
PRISMA FLOW diagram for study selection

Our search identified a total of 3774 records (Fig. 1). Out of these, 1873 were retrieved through Pubmed via MEDLINE, 696 through EMBASE, 922 through CENTRAL and 283 through CINAHL and PsychInfo. An additional 10 records were identified through other sources (identified by manually searching the reference list of included studies). After removal of duplicates, we screened 2517 records and identified 101 full text articles for eligibility assessment. Of these, 33 studies were included in the narrative synthesis. Studies reported various outcomes: weight (*n* = 16) [32–47], BMI (*n* = 19) [32–37, 39–44, 46, 48–53], waist circumference (*n* = 13) [32–36, 39, 42, 43, 45–47, 51, 54], lipids (*n* = 15) [32, 34–37, 39, 42, 44–47, 49, 51, 52, 55], triglycerides (*n* = 8) [37, 39, 44–47, 49, 52], blood pressure (n = 16) [32–37, 39, 42–47, 49, 51, 52] and glucose (*n* = 10) [32, 34, 37, 39, 44–47, 49, 52]. A total of 24 studies were included in the meta-analysis. Data from other studies was not available.

Common reasons for excluding studies from the review are reported in the PRISMA diagram. Twelve RCTs [32–39, 49, 54, 56, 57], 15 cluster RCTs [40, 41, 43, 44, 46–48, 50, 53, 58–63], 3 quasi experimental trials [42, 52, 64], and 3 controlled trials [45, 51, 55] were included in the review. A total of 36,188 men and women aged 32 to 55 years participated in these studies with the study sample sizes ranging from 45 to 10,281.

The descriptive characteristics of the included studies are presented in Table 1. The studies had a varied population which included school and university personnel, employees of public and private sectors, blue collar workers (carpenters, bricklayers, road workers, crane operators, locomotive maintenance workers, gardeners, drivers, transportation workers, garage staff and factory workers), professional and technical, salaried and hourly workers, hospital staff, security guards, healthcare workers, casino employees and industry workers.

**Table 1:** Descriptive characteristics of the included studies

Author	Year of Publication	Country	Study Design	Study Duration	Study Setting	Study Population	Sample Size	Age	Gender	Study Outcomes
Almeida et al	2015	USA	cluster RCT	18 months	Worksites in Virginia	Worksite employees with a BMI > =25	1790	46.9 (3.2)	Females (73.8%)	Body weight (kg), BMI (kg/m^2^); measured at 6 months
Atlantis et al	2006	Australia	RCT	1 year	Australian Casino	Healthy but sedentary casino subjects	73	32 (8)	Males (48%)	Waist circ (cm); measured at 6 months
Barham et al	2011	USA	RCT	19 months	Onondaga county NY	Pre-diabetic or diabetic employees at the county	45	51.2 (8.0)	Males (16%)	Body weight (kg), BMI (kg/m^2^), waist circ (cm), blood pressure (mm Hg), lipids (mg/dl), glucose (mg/dl); measured at 6 and 12 months
Brehm et al	2009	US	cluster RCT	–	8 manufacturing companies	manufacturing company employees	341	43.8 (10.0)	Males (60%)	Body weight (kg), blood pressure (mm Hg), lipids (mg/dl), glucose (mg/dl); measured at 6 and 12 months
Chen et al	2016	Taiwan	quasi experimental	24 weeks	3 worksites in Taiwan	Full time older industrial workers	108	54.5 (3.7), 55.7 (4.0)	Males (39.7, 52%)	Body weight (kg), BMI (kg/m^2^), waist circ (cm), blood pressure (mm Hg), lipids (mg/dl); measured at 6 months
Chockalingam et al	2008	Canada	RCT	–	Employees in the Halifax area, Nova Scotia	Employees with at least 2 modifiable coronary risk factors	397	44 (8)	Males (51%)	BMI (kg/m^2^), lipids (mg/dl), blood pressure (mg/dl); measured at 3 and 6 months
Christensen et al	2012	Denmark	cluster RCT	14 months	Danish Municipality in central Jutland	Female overweight health care workers	98	–	Females (100%)	Body weight (kg), BMI (kg/m^2^), waist circ (cm), blood pressure (mm Hg); measured at 12 months
Engbers et al	2007	Netherlands	controlled trial	1 year	2 government companies	overweight office employees with a BMI > =23	540	45.3 (9.6), 45.5 (8.7)	Females (37.4, 41.7%)	BMI (kg/m^2^), waist circ (cm), lipids (mg/dl), blood pressure (mm Hg); measured at 12 months
Fernandez et al	2015	USA	cluster RCT	5 years	nonunionized manufacturing, R&D company with multiple sites in the northeastern United States	Worksite employees	3799	47.7 (7.4), 47.4(7.8)	Males(68.1, 55.6)	BMI (kg/m^2^); measured at 36 months
French et al	2010	Minneapolis	cluster RCT	2 years	4 garages; 2 urban, 2 suburban	garage workers	832	49	Males (79%)	BMI (kg/m^2^); measured at 18 months
Goetzel et al	2009	USA	quasi experimental	1 year	12 sites of Dow science and technology company	All employees in the manufacturing, r&d and administration departments at all sites	10,281	44.3, 44.1	Males (26.7, 25%)	Body weight (kg), BMI (kg/m^2^), blood pressure (mm Hg), lipids (mg/dl), glucose (mg/dl); measured at 12 months
Healy et al	2017	Australia	Cluster RCT	4 years	Worksites from a large public service organization	Worksite employees	231	45.6 (9.4)	Males (32%)	Body weight (kg), waist (cm), blood pressure (mm Hg), lipids (mg/dl), glucose (mg/dl); measured at 12 months
Jamal et al	2016	Malaysia	RCT	2 years	Melbourne	Overweight/obese employees from a local university	194	40.5 (9.3)	Women (72.7%)	Body weight (kg), BMI (kg/m^2^), waist circ (cm), blood pressure (mm Hg), lipids (mg/dl), glucose (mg/dl); measured at 6 months
Kim et al	2015	Korea	RCT	6 months	3 Korean worksites	Employees from the Korean gas corporation, district heating corporation and expressway corporation with a BMI > 25 kg/m2	196	41.02 (6.82), 41.5 (6.98)	Males (100%)	Body weight (kg); measured at 6 months
Kramer et al	2015	USA	RCT	18 months	Bayer corporation in Pittsburgh	Pre diabetic employees both professional and technical, salaried and hourly workers with BMI > =24	89	52.3 (7.2)	Males (45%)	Body weight (kg), BMI (kg/m^2^), waist circ (cm), blood pressure (mm Hg), lipids (mg/dl), glucose (mg/dl); measured at 6 months
Lemon et al	2010	USA	cluster RCT	3 years	6 hospitals in massachussets	Hospital employees	806	–	Males (19%)	BMI (kg/m^2^); measured at 12 and 24 months
Lemon et al	2014	USA	cluster RCT	3 years	12 central Massachusetts public high schools	School employees	782	–	Males (33%)	Body weight (kg), BMI (kg/m^2^); measured at 12 and 24 months
Limaye et al	2016	India	RCT	3 years	two multinational IT industries in Pune	Employees with ≥3 risk factors (family history of CVD, obesity, highblood pressure, impaired glucose, impaired lipids)	265	36.8 (7.2), 35.7 (8.1)	Males (74, 71%)	Body weight (kg), BMI (kg/m^2^), waist circ (cm), blood pressure (mm Hg), lipids (mg/dl), glucose (mg/dl); measured at 12 months
Linde et al	2012	USA	cluster RCT	3 years	Six worksites in the Twin cities area Minnesota	Worksite employees	1672	–	Males (39.3%)	BMI (kg/m^2^); measured at 24 months
Milani et al	2009	USA	cluster RCT	1 year	2 geographically disparate work locations of a single employer	Worksite employees	339	40 (8), 43 (10)	Males (52, 53%)	Body weight (kg), lipids (mg/dl), blood pressure (mm Hg), glucose (mg/dl); measured at 6 months
Morgan et al	2011	Australia	RCT	14 weeks	Tomago Aluminium company	Over-weight/obese male shift workers	110	44.4 (8.6)	Males (100%)	Body weight (kg), BMI (kg/m^2^), waist circ (cm), blood pressure (mm Hg); measured at 14 months
Moy et al	2006	Malaysia	quasi experimental	2 years	public health university and teaching hospital in KL	Security guards	186	45.6 (7.2), 48 (4.7)	Males (100%)	BMI (kg/m^2^), lipids (mg/dl), blood pressure (mm Hg), glucose (mg/dl); measured at 24 months
Muto et al	2001	Japan	RCT	18 months	building maintenance company in Japan	Building maintenance company workers with at least one abnormal CVD risk factor	352	42.3 (4.5), 42.7 (2.7)	Males (100%)	Body weight (kg), BMI (kg/m^2^), lipids (mg/dl), blood pressure (mm Hg), glucose (mg/dl); measured at 18 months
Naito et al	2008	Japan	controlled trial	5 years	Factories in Japan	Factory employees	2929	44.2 (8), 39.5 (7.6)	–	HDL (mg/dl); measured at 60 months
Nilsson et al	2001	Sweden	RCT	18 months	4 branches of helsingborg public sector	Nurses, cleaners, gardeners, drivers or transportation workers with a CVD risk score greater than 9	89	49.7		BMI (kg/m^2^), lipids (mg/dl), blood pressure (mm Hg), glucose (mg/dl); measured at 18 months
Prabhakaran et al	2009	India	controlled trial	4 years	Industrial sites in India	industry employees	6889	40.8 (10.8), 38.6 (11.7)	Males (58.7%, 58.1)	Body weight (kg), waist circ (cm), lipids (mg/dl), blood pressure (mm Hg), glucose (mg/dl); measured at 48 months
Racette et al	2009	USA	cluster RCT	1 year	Worksites within a large medical center in Missouri	Medical centre employees aged 18 and above	123	45 (9)	Males (11.25)	Body weight (kg), BMI (kg/m^2^), lipids (mg/dl), blood pressure (mm Hg), glucose (mg/dl); measured at 12 months
Siegel et al	2010	USA	cluster RCT	2 years	16 elementary schools in 2 areas of LA	All school employees	413	40 (0.80)	Males (17%)	BMI (kg/m2); measured at 2 years
Shrivastava et al	2017	India	cluster RCT	6 months	4 worksites from Delhi-NCR	overweight employees	267	35.8 (7.6), 39 (8.7)	Males (87.9%)	Body weight (kg), BMI (kg/m^2^), waist circ (cm), blood pressure (mm Hg), lipids (mg/dl), glucose (mg/dl); measured at 6 months
Viester et al	2017	Netherlands	RCT	12 months	Construction company in Netherlands	Blue collar workers (carpenters, road workers, crane operators,and factory workers.)	314	47 (9.5)	–	Body weight (kg), BMI (kg/m^2^), waist circ (cm), blood pressure (mm Hg), lipids (mg/dl); measured at 12 months
Weinhold et al	2015	USA	RCT	2 years	University in US	Worksite pre-diabetic employees with a BMI more than 25	69	51.6 (9.5), 51.0 (8.1)	Males (20, 20.6%)	Body weight (kg), BMI (kg/m2), waist circ (cm) blood pressure (mm Hg); measured at 7 months
Williams et al	2014	USA	cluster RCT	2 years	30 Hotels in Hawaii	Hotel employees with a BMI > =25	1207	46 (9.6), 46.1 (10.2)	Males (49.8, 46.6%)	BMI (kg/m^2^); measured at 12 and 24 months
Wilson et al	2016	USA	cluster RCT	12 months	Railroad maintenance facilities of Union Pacific Railroad	Locomotive maintenance employees at the company	362	47, 44	Males (93.7, 94.6%)	Body weight (kg), BMI (kg/m^2^); measured at 12 months

Out of the 33 studies reviewed, 13 studies [32–34, 36, 38–40, 43, 46, 49, 51, 56, 60] (8 RCTs, 4 cluster RCTs and 1 controlled trial) included only employees who had at least one raised CVD risk factor while the other 20 studies [35, 37, 41, 42, 44, 45, 47, 48, 50, 52–55, 57–59, 61–64] included all employees irrespective of their health status.

### Narrative analysis

#### Study interventions

The studies used different types of interventions like campaigns, workshops and education; individual level behavioral change; and changes to the office environment and policies. Out of the 33 studies reviewed, 28 studies [32, 36, 37, 39–47, 49–64] used a mix of the three approaches whereas the other 5 studies [33–35, 38, 48] implemented any one of these three approaches. The intervention duration in all the studies ranged from 6 months to 5 years. Campaign approach included lifestyle coaches to educate on physical activity, workshops on cardiac risk factors, wellness fairs, point of choice prompts and information dissemination through newsletters, brochures, internet etc. Behavioral change included incentivized group activities or tailored-for-individual weight loss regimes through physical activity, goal setting and rewards. Organizational changes included making stairs and walls more aesthetic, mapping of walking routes and more. Detailed description of the intervention and control groups is presented in Additional File 2.

#### Risk of bias in included studies

**Fig. 2:**
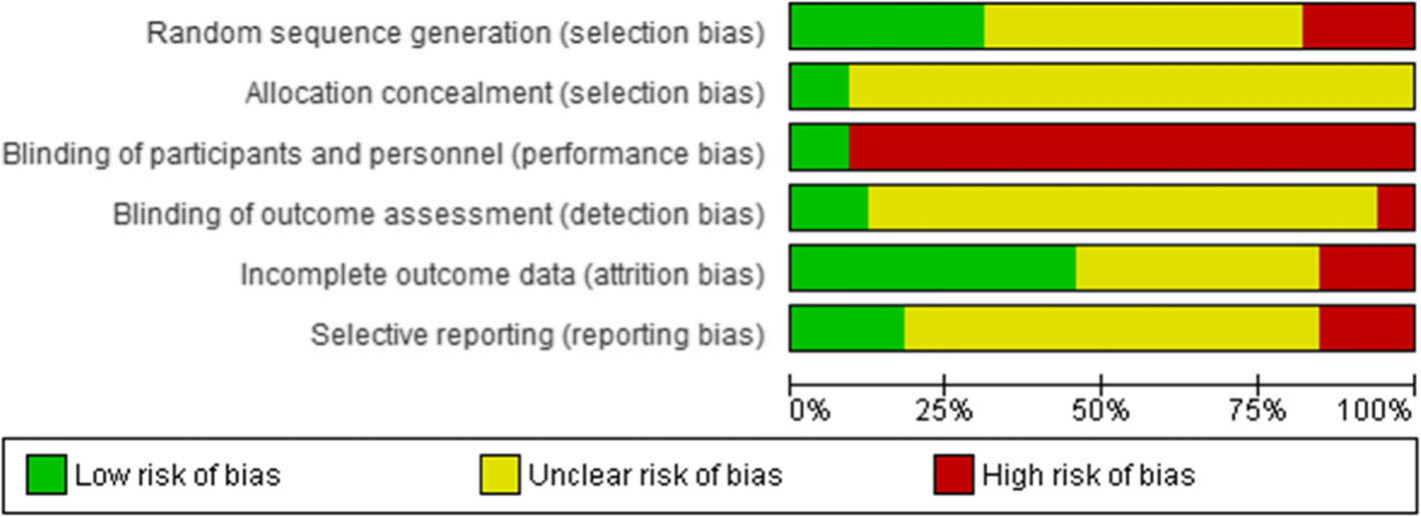
Risk of bias graph- review authors’ judgments about each risk of bias item presented as percentages across all included studies

Risk of bias among the included studies was assessed using the Cochrane risk of bias assessment tool as shown in Fig. 2. The risk of bias summary for individual studies has been presented in Additional File 4.

The highest risk of bias emanated from performance bias due to unblinded participants and study personnel. There was also a high unclear risk of selection bias and detection bias due to lack of adequate data reported on randomization, allocation concealment and blinding of study outcome assessors.

### Meta-analysis

#### Intervention effects on cardio-metabolic risk markers

We undertook exploratory meta-analyses to pool the effect estimates for body weight, body mass index, waist circumference, systolic and diastolic blood pressure, total cholesterol, low density lipoprotein (LDL-C) and high density lipoprotein (HDL-C) cholesterol, triglycerides and blood glucose. Review Manager Software (RevMan version 5.3) was used to generate forest plots. The random effects model was used to generate intervention effects.

Results from the meta-analyses showed an overall significant intervention effect for **body weight** (16 studies, Mean difference: -2.61, 95% CI- -3.89, − 1.33), **body mass index** (19 studies, Mean difference: -0.42, 95% CI- -0.69, − 0.15) and **waist circumference** (13 studies, Mean difference: -1.92, 95% CI- -3.25, − 0.60) but there was considerable heterogeneity among estimates (I^2^ = 94, 89 and 92% respectively; *p*-value < 0.0001). The pooled estimates for lipids, blood pressure and blood glucose were not statistically significant.

The overall mean difference and 95% CIs for each outcome, along with the heterogeneity in individual studies have been presented in Table 2. Exploratory sub-group analysis showed a significant sub-group effect by study design for body weight (*p* = 0.0008) and BMI (*p* < 0.00001) and by intervention type for BMI (*p* = 0.008) and TC (*p* = 0.0007). However, there was no sub-group effect for the other outcomes (waist circumference and biochemical markers). (Additional File 3) In conclusion, sub-groups could not explain the high levels of heterogeneity responsible for the variability in study effect size estimates because the I-squared values were not reduced substantially.

**Table 2:** Pooled estimates from meta-analysis of studies for change in each CVD risk outcome

Outcome	Number of studies	Mean difference	Confidence interval	Heterogeneity
Body weight (kg)	16	**-2.61**	**[−3.89, − 1.33]**	94%
Body mass index (kg/m^2^)	19	**-0.42**	**[−0.69, − 0.15]**	89%
Waist circumference (cm)	13	**-1.92**	**[−3.25, −0.60]**	92%
Systolic blood pressure (mmHg)	16	−1.73	[−4.25, 0.79]	93%
Diastolic blood pressure (mmHg)	15	−1.73	[−4.25, 0.79]	93%
Total cholesterol (mg/dl)	11	−3.75	[−9.84, 2.33]	86%
HDL cholesterol (mg/dl)	12	0.54	[−1.13, 2.20]	88%
LDL cholesterol (mg/dl)	10	−3.25	[−8.00, 1.51]	75%
Triglycerides (mg/dl)	8	0.62	[−4.82, 6.06]	55%
Blood glucose (mg/dl)	10	−3.14	[−6.47, 0.20]	94%

Estimates highlighted in bold indicate the effect sizes that were statistically significant

Also, since these analyses usually involve multiple testing in case of many outcomes and would ideally require a much smaller *p*-value cut-off for significance, sub-group analysis estimates are observational and should be interpreted with caution.

The forest plots for all the individual outcomes have been shown in the figures below. Each forest plot shows the individual effect estimates for the intervention and control groups and the mean difference in each study, along with the overall pooled mean difference and corresponding CIs. (Figs. 3, 4, 5, 6, 7, 8, 9, 10, 11, 12).

**Fig. 3:**
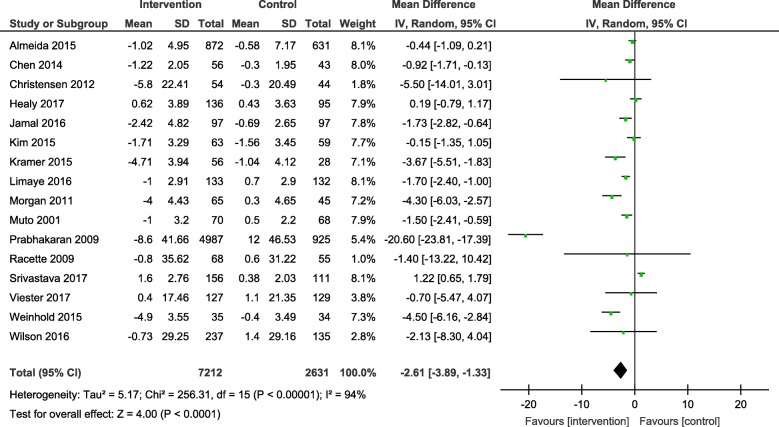
Forest plot for change in body weight

**Fig. 4:**
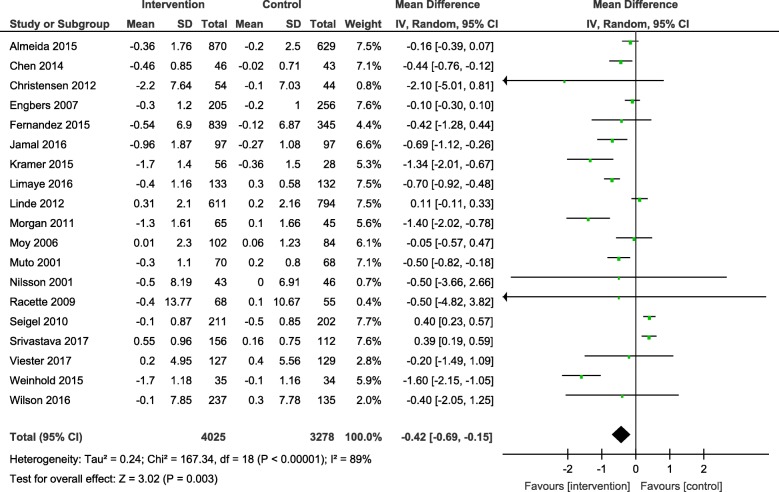
Forest plot for change in body mass index

**Fig. 5:**
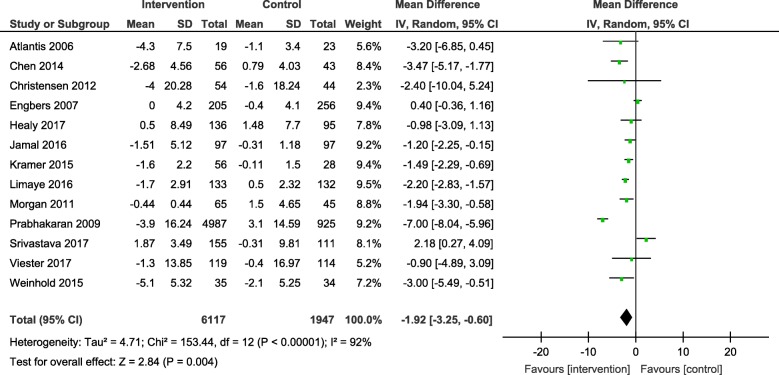
Forest plot for change in waist circumference

**Fig. 6:**
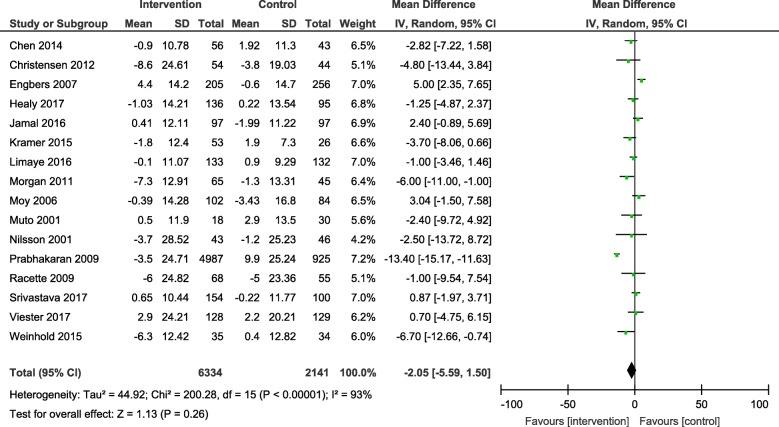
Forest plot for change in systolic blood pressure

**Fig. 7:**
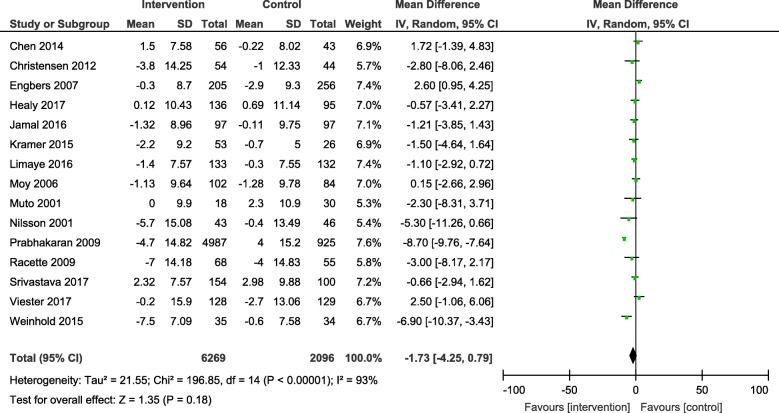
Forest plot for change in diastolic blood pressure

**Fig. 8:**
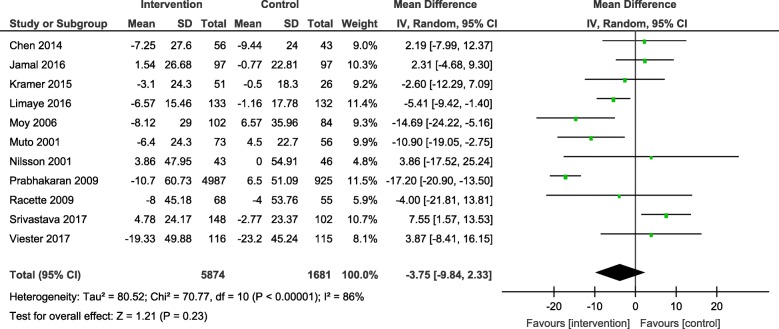
Forest plot for change in total cholesterol

**Fig. 9:**
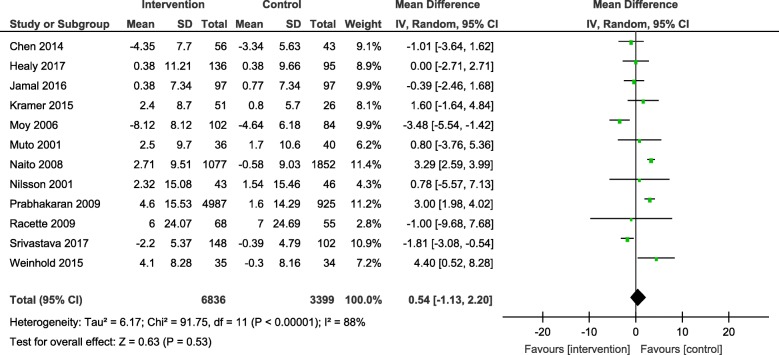
Forest plot for change in HDL-cholesterol

**Fig. 10:**
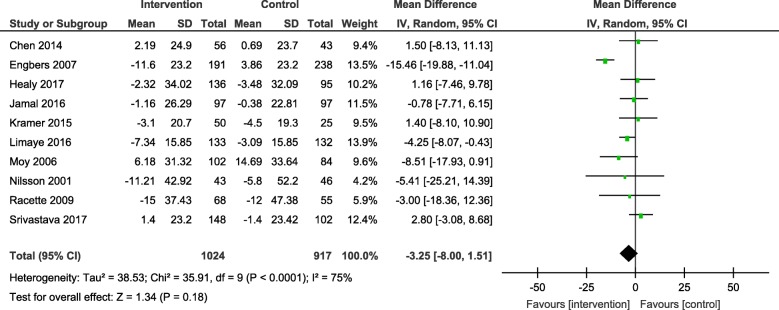
Forest plot for change in LDL-cholesterol

**Fig. 11:**
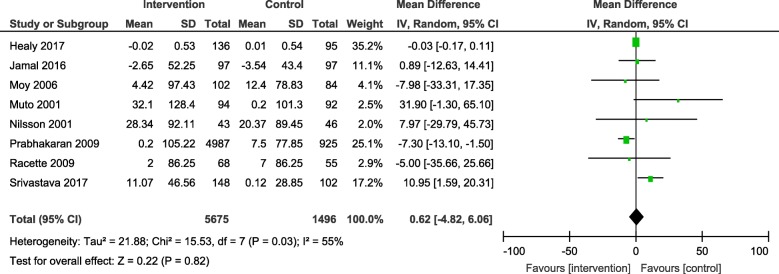
Forest plot for change in triglycerides

**Fig. 12:**
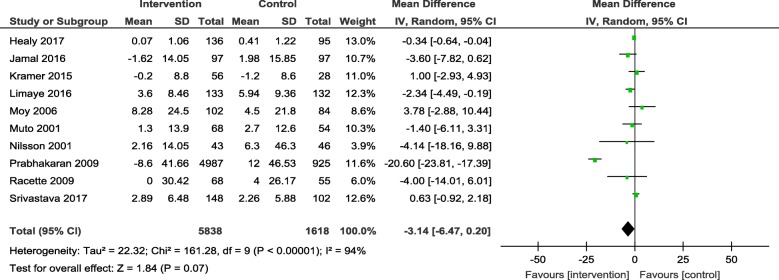
Forest plot for change in blood glucose

(A visual assessment of the funnel plots for each outcome showed the presence of some asymmetry for a few biochemical outcomes but we did not conduct any formal statistical tests to assess the same.)

## Discussion

Based on the 33 studies reviewed, we found that changes in diet and physical activity at worksites had a significant and positive effect on body weight, body mass index and waist circumference of working adults. It can be concluded that workplace based physical activity interventions can positively affect anthropometric outcomes and thus have the potential to alter the biochemical risk markers too. The results need to be interpreted with caution though, due to high heterogeneity among studies. The *p*-values for the chi-square test for heterogeneity were quite significant, suggesting a high degree of variability in effect estimates due to actual differences in studies and not due to sampling error (chance). This may be due to variability in sample sizes (they ranged from 45 to 10,281 participants) as well as the different study designs used in different studies. The various intervention approaches used across studies might also have contributed to the heterogeneity, as indicated by the exploratory sub-group analyses.

There could be a few reasons for the lack of a stronger evidence for the effect on biochemical variables. Anthropometric outcomes were the primary outcomes in almost all the studies whereas biochemical outcomes *in a third, and* only as secondary outcomes in half of the studies included in the meta-analyses. Those studies were therefore not adequately powered to detect significant changes in blood pressure, lipids and glucose levels.

Some reviews done in the past have shown a similar pattern with most included studies focusing only on anthropometric outcomes, which underscores the need for more high quality trials studying the effect of physical activity interventions on blood pressure and biochemical measures as well [65] [66]**.**

A few previously done reviews such as the one by Fleming et al. [67]**,** a 2010 review by Groeneveld et al. [26] and a brief overview of worksite health promotion programs and non-communicable disease prevention [68] have all suggested the possibility of greater intervention effectiveness among populations already at risk of CVDs compared to mixed populations. Hence, there is need for better quality studies to ascertain the role of employee health status in intervention effectiveness. A comparison of the effects of individual level behavior change on CVD risk reduction, compared to educational approaches and changes to the office environment is also an interesting facet that can be further explored.

Another aspect that needs consideration is participant compliance and barriers to intervention adherence. Unlike clinical or medical interventions which can be constantly monitored for acceptability, the effectiveness of lifestyle based interventions is difficult to evaluate since intervention uptake is a complex measure [69]. Some studies concluded lack of compliance, issues with intervention adherence, low participation and retention rates and inadequately motivated employees as some of the reasons which could have affected the study results. Non-adherence could also be one reason for very small effect sizes in studies with larger sample sizes [70].

Long-term participation and employee adherence thus seem to be major challenges in implementation of worksite physical activity interventions [70] [71]. It becomes paramount to devise innovative and practical ways to motivate the workforce and ensure sustained interest of the participants throughout the study [72]. Greater adherence and acceptability would ensure greater uptake that would in-turn result in more tangible health benefits to the employees.

### Limitations and strengths

Our review has a few strengths. To the best of our knowledge, this is the first meta-analysis focused solely on the anthropometric and biochemical outcomes related to physical activity interventions at worksite. Secondly, the last review reporting the effects of worksite interventions on anthropometric and biochemical CVD risk markers was done in 2010 and our work provides updated literature on the topic. Thirdly, considering that we were dealing with multi-component PA interventions with multiple outcomes (and not a drug trial) we used a broad search strategy and covered 5 different databases to obtain a synthesis of all the relevant literature for practical understanding and future research. Fourthly, unlike a majority of previous reviews assessing the effect of worksite PA interventions primarily on physical activity, the proximal outcome, our review goes to the next level and summarizes the effects on the more distant anthropometric and biochemical outcomes.

A limitation of our study was that assessment of bias in individual studies was based on the data as reported in them. In some studies, relevant information on aspects of randomization and reporting of data was not presented which may have led to an underestimation of their quality. Another limitation was that we could not include data from nine studies in our meta-analyses since the estimates required for the analysis were not available. We wrote to the study authors but unfortunately only one of them provided data for our analyses. Additionally, it is possible that the interventions caused a change in other health behaviors like diet too, apart from physical activity, which in-turn could have led to an improvement in CVD outcomes.

## Conclusions

Worksite physical activity interventions were effective in improving anthropometric measures, namely body weight, BMI and waist circumference. We were however unable to demonstrate a significant effect on biochemical variables. A possible reason could be that *almost two-third* of the studies *were either not* reporting the biochemical outcomes or not adequately powered to assess intervention effects on these variables. The potential of such interventions to prevent CVD and overall non-communicable diseases (NCDs) needs attention by employers and policy makers for improving the health status of the population. This can significantly contribute to achieving the UN targets of a 25% relative reduction in premature deaths from NCDs by 2025 [73].

### Implications for future research

Overall, the evidence on the wide-ranging benefits of physical activity interventions is robust for action, and the absence of statistically significant biochemical improvements should not act as a deterrent to adoption by worksites. Ways to enhance uptake of worksite physical activity interventions by employers, employees and the environment need to be studied. A robust process evaluation framework along with assessment of factors like dietary changes, frequency of sickness, back pain, absenteeism etc., would provide greater insights into the relative effectiveness and complementarity of the different types of interventions. A design based on a theoretical framework like the Medical Research Council framework [74] for designing and evaluating complex intervention studies is an option. Also, future worksite PA intervention studies should adequately power for the biochemical outcomes and have longer follow-up durations. Hard-endpoints should be strived for wherever possible.

## Supplementary information

**Additional file 1.** Search terms. This file provides the search strategy used to obtain relevant articles from PUBMED.

**Additional file 2.** Study interventions. This file includes a table that describes the purpose, characteristics, interventions and results of each study included in the review.

**Additional file 3.** Exploratory Sub-group analyses. This file includes the tables describing the effects of workplace interventions on outcomes, analyzed under sub-groups.

**Additional file 4.** Risk of bias summary for individual studies in the review.

## Data Availability

Not Applicable

## Abbreviations

BMI: Body Mass Index
CI: Confidence Interval
CVD: Cardiovascular Disease
HDL-C: High Density Lipoprotein Cholesterol
LDL-C: Low Density Lipoprotein Cholesterol
MVPA: Moderate to Vigorous Physical Activity
PA: Physical Activity
RCT: Randomized Controlled Trial
WHO: World Health Organization

## References

[1] Yusuf S, Hawken S, Ôunpuu S, Dans T, Avezum A, Lanas F (2004). Effect of potentially modifiable risk factors associated with myocardial infarction in 52 countries (the INTERHEART study): case-control study. Lancet.

[2] Lee I-M, Shiroma EJ, Lobelo F, Puska P, Blair SN, Katzmarzyk PT (2012). Impact of physical inactivity on the World’s major non-communicable diseases. Lancet.

[3] WHO | Physical Inactivity: A Global Public Health Problem [Internet]. WHO. 2017 [cited 2017 Dec 27]. Available from: http://www.who.int/dietphysicalactivity/factsheet_inactivity/en/

[4] Diabetes mellitus and exercise [Internet]. [cited 2018 Jul 9]. Available from: http://care.diabetesjournals.org/content/diacare/25/suppl_1/s64.full.pdf

[5] 2018 Physical Activity Guidelines Advisory Committee Scientific Report. 2018;779.

[6] Andajani-Sutjahjo S, Ball K, Warren N, Inglis V, Crawford D (2004). Perceived personal, social and environmental barriers to weight maintenance among young women: a community survey. Int J Behav Nutr Phys Act.

[7] Anjali SM (2018). Perceived barriers of young adults for participation in physical activity. Curr Res Nutr Food Sci J.

[8] Trost SG, Owen N, Bauman AE, Sallis JF, Brown W (2002). Correlates of adults’ participation in physical activity: review and update. Med Sci Sports Exerc.

[9] van Uffelen JGZ, Wong J, Chau JY, van der Ploeg HP, Riphagen I, Gilson ND (2010). Occupational sitting and health risks: a systematic review. Am J Prev Med.

[10] Tudor-Locke C, Leonardi C, Johnson WD, Katzmarzyk PT (2011). Time spent in physical activity and sedentary behaviors on the working day: the American time use survey. J Occup Environ Med.

[11] Jirathananuwat A, Pongpirul K (2017). Promoting physical activity in the workplace: a systematic meta-review. J Occup Health.

[12] Phillips D. A settings approach: Healthy@Work—a model of a health promoting workplace. :7.

[13] The Settings Approach: A Guiding Framework for HECA [Internet]. [cited 2018 Jul 9]. Available from: http://www.who.int/heca/alliancebuilding/heca2wg_draftsettings_180303.PDF

[14] Proper K, van Mechelen W. Effectiveness and economic impact of worksite interventions to promote physical activity and healthy diet. :63.

[15] Worksite Physical Activity | Physical Activity | CDC [Internet]. 2019 [cited 2019 Sep 15]. Available from: https://www.cdc.gov/physicalactivity/worksite-pa/index.htm

[16] Pronk NP (2009). Physical activity promotion in business and industry: evidence, context, and recommendations for a national plan. J Phys Act Health.

[17] Chu AHY, Ng SHX, Tan CS, Win AM, Koh D, Müller-Riemenschneider F (2016). A systematic review and meta-analysis of workplace intervention strategies to reduce sedentary time in white-collar workers. Obes Rev Off J Int Assoc Study Obes.

[18] Shrestha N, Harjula K, Verbeek J, Ijaz S. Workplace interventions for reducing sitting at work - Shrestha - 2016 - The Cochrane Library - Wiley Online Library [Internet]. 2017 [cited 2017 Aug 17]. Available from: http://onlinelibrary.wiley.com/doi/10.1002/14651858.CD010912.pub3/full

[19] Hutcheson AK, Piazza AJ, Knowlden AP. Work site–based environmental interventions to reduce sedentary behavior: a systematic review. Am J Health Promot. 2016:0890117116674681. 10.1177/0890117116674681 27780893

[20] Reed JL, Prince SA, Elliott CG, Mullen K-A, Tulloch HE, Hiremath S, et al. Impact of Workplace Physical Activity Interventions on Physical Activity and Cardiometabolic Health Among Working-Age Women: A Systematic Review and Meta-Analysis. Circ Cardiovasc Qual Outcomes. 2017;10(2). 10.1161/CIRCOUTCOMES.116.003516 28228457

[21] Commissaris DA, Huysmans MA, Mathiassen SE, Srinivasan D, Koppes LL, Hendriksen IJ (2016). Interventions to reduce sedentary behavior and increase physical activity during productive work: a systematic review. Scand J Work Environ Health.

[22] Plotnikoff R, Collins CE, Williams R, Germov J, Callister R (2015). Effectiveness of interventions targeting health behaviors in university and college staff: a systematic review. Am J Health Promot AJHP.

[23] Chen TTL, Magnussen CG, To QG, To KG (2013). Workplace physical activity interventions: a systematic review. Am J Health Promot AJHP..

[24] Barr-Anderson DJ, AuYoung M, Whitt-Glover MC, Glenn BA, Yancey AK (2011). Integration of short bouts of physical activity into organizational routine a systematic review of the literature. Am J Prev Med.

[25] Chau Josephine Y., der Ploeg Hidde P. van, van Uffelen Jannique G.Z., Wong Jason, Riphagen Ingrid, Healy Genevieve N., Gilson Nicholas D., Dunstan David W., Bauman Adrian E., Owen Neville, Brown Wendy J. (2010). Are workplace interventions to reduce sitting effective? A systematic review. Preventive Medicine.

[26] Groeneveld IF, Proper KI, van der Beek AJ, Hildebrandt VH, van Mechelen W (2010). Lifestyle-focused interventions at the workplace to reduce the risk of cardiovascular disease--a systematic review. Scand J Work Environ Health.

[27] Prospero Review Protocol [Internet]. [cited 2018 Jul 9]. Available from: https://www.crd.york.ac.uk/prospero/display_record.php? RecordID=94436.

[28] DerSimonian R, Laird N (1986). Meta-analysis in clinical trials. Control Clin Trials.

[29] 9.5.2 Identifying and measuring heterogeneity [Internet]. [cited 2019 Sep 16]. Available from: https://handbook-5-1.cochrane.org/chapter_9/9_5_2_identifying_and_measuring_heterogeneity.htm

[30] Heath GW, Parra DC, Sarmiento OL, Andersen LB, Owen N, Goenka S (2012). Evidence-based intervention in physical activity: lessons from around the world. Lancet Lond Engl.

[31] Assessing Risk of Bias in Included Studies [Internet]. [cited 2018 Aug 6]. Available from: https://handbook-5-1.cochrane.org/chapter_8/8_assessing_risk_of_bias_in_included_studies.htm.

[32] Kramer M, Molenaar D, Arena V, Venditti E, Meehan R, Miller R (2015). Improving employee health: evaluation of a worksite lifestyle change program to decrease risk factors for diabetes and cardiovascular disease. J Occup Environ Med Am Coll Occup Environ Med.

[33] Morgan PJ, Collins CE, Plotnikoff RC, Cook AT, Berthon B, Mitchell S (2011). Efficacy of a workplace-based weight loss program for overweight male shift workers: the workplace POWER (preventing obesity without eating like a rabbit) randomized controlled trial. Prev Med.

[34] Limaye T, Kumaran K, Joglekar C, Bhat D, Kulkarni R, Nanivadekar A (2017). Efficacy of a virtual assistance-based lifestyle intervention in reducing risk factors for type 2 diabetes in young employees in the information technology industry in India: LIMIT, a randomized controlled trial. Diabet Med J Br Diabet Assoc.

[35] Viester L, Verhagen EALM, Bongers PM, van der Beek AJ (2017). Effectiveness of a worksite intervention for male construction workers on dietary and physical activity behaviors, body mass index, and health outcomes: results of a randomized controlled trial. Am J Health Promot AJHP..

[36] Weinhold KR, Miller CK, Marrero DG, Nagaraja HN, Focht BC, Gascon GM. A Randomized Controlled Trial Translating the Diabetes Prevention Program to a University Worksite, Ohio, 2012–2014. Prev Chronic Dis [Internet]. 2015 Nov 25 [cited 2017 Dec 12];12. Available from: https://www.ncbi.nlm.nih.gov/pmc/articles/PMC4674443/10.5888/pcd12.150301 PMC4674443 26605710

[37] Muto T, Yamauchi K (2001). Evaluation of a multicomponent workplace health promotion program conducted in Japan for improving employees’ cardiovascular disease risk factors. Prev Med.

[38] Kim J-Y, Oh S, Steinhubl S, Kim S, Bae WK, Han JS, et al. Effectiveness of 6 Months of Tailored Text Message Reminders for Obese Male Participants in a Worksite Weight Loss Program: Randomized Controlled Trial. JMIR MHealth UHealth [Internet]. 2015 Feb 3 [cited 2017 Dec 12];3(1). Available from: https://www.ncbi.nlm.nih.gov/pmc/articles/PMC4342743/10.2196/mhealth.3949 PMC4342743 25648325

[39] Jamal SN, Moy FM, Mohamed MNA, Mukhtar F (2016). Effectiveness of a group support lifestyle modification (GSLiM) Programme among obese adults in workplace: a randomised controlled trial. PLoS One.

[40] Almeida FA, You W, Harden SM, Blackman KCA, Davy BM, Glasgow RE (2015). Effectiveness of a worksite-based weight loss randomized controlled trial: the WORKSITE study. Obes Silver Spring Md.

[41] Wilson MG, DeJoy DM, Vandenberg R, Padilla H, Davis M (2016). FUEL your life: a translation of the diabetes prevention program to worksites. Am J Health Promot AJHP..

[42] Chen M-M, Tsai AC, Wang J-Y (2016). The effectiveness and barriers of implementing a workplace health promotion program to improve metabolic disorders in older workers in Taiwan. Glob Health Promot.

[43] Christensen J, Overgaard K, Carneiro I, Holtermann A, Søgaard K (2012). Weight loss among female health care workers- a 1-year workplace based randomized controlled trial in the FINALE-health study. BMC Public Health.

[44] Racette SB, Deusinger SS, Inman CL, Burlis TL, Highstein GR, Buskirk TD (2009). Worksite opportunities for wellness (WOW): effects on cardiovascular disease risk factors after 1 year. Prev Med.

[45] Prabhakaran D, Jeemon P, Goenka S, Lakshmy R, Thankappan KR, Ahmed F (2009). Impact of a worksite intervention program on cardiovascular risk factors: a demonstration project in an Indian industrial population. J Am Coll Cardiol.

[46] Shrivastava U, Fatma M, Mohan S, Singh P, Misra A. Randomized Control Trial for Reduction of Body Weight, Body Fat Patterning, and Cardiometabolic Risk Factors in Overweight Worksite Employees in Delhi, India. J Diabetes Res [Internet]. 2017 [cited 2018 9];2017. Available from: https://www.ncbi.nlm.nih.gov/pmc/articles/PMC5727835/10.1155/2017/7254174 PMC5727835 29318159

[47] Healy GN, Winkler EAH, Eakin EG, Owen N, Lamontagne AD, Moodie M (2017). A cluster RCT to reduce workers’ sitting time: impact on Cardiometabolic biomarkers. Med Sci Sports Exerc.

[48] Siegel JM, Prelip ML, Erausquin JT, Kim SA (2010). A worksite obesity intervention: results from a group-randomized trial. Am J Public Health.

[49] Nilsson PM, Klasson EB, Nyberg P (2001). Life-style intervention at the worksite--reduction of cardiovascular risk factors in a randomized study. Scand J Work Environ Health.

[50] Fernandez ID, Chin NP, Devine CM, Dozier AM, Martina CA, McIntosh S (2015). Images of a healthy worksite: a group-randomized trial for worksite weight gain prevention with employee participation in intervention design. Am J Public Health.

[51] Engbers LH, van Poppel MNM, van Mechelen W (2007). Modest effects of a controlled worksite environmental intervention on cardiovascular risk in office workers. Prev Med.

[52] Moy F, Sallam AAB, Wong M (2006). The results of a worksite health promotion programme in Kuala Lumpur. Malaysia Health Promot Int.

[53] Linde JA, Nygaard KE, MacLehose RF, Mitchell NR, Harnack LJ, Cousins JM (2012). HealthWorks: results of a multi-component group-randomized worksite environmental intervention trial for weight gain prevention. Int J Behav Nutr Phys Act.

[54] Atlantis E, Chow C-M, Kirby A, Fiatarone Singh MA (2006). Worksite intervention effects on physical health: a randomized controlled trial. Health Promot Int.

[55] Naito M, Nakayama T, Okamura T, Miura K, Yanagita M, Fujieda Y (2008). Effect of a 4-year workplace-based physical activity intervention program on the blood lipid profiles of participating employees: the high-risk and population strategy for occupational health promotion (HIPOP-OHP) study. Atherosclerosis..

[56] Barham K, West S, Trief P, Morrow C, Wade M, Weinstock RS (2011). Diabetes prevention and control in the workplace: a pilot project for county employees. J Public Health Manag Pract JPHMP.

[57] Chockalingam A, Kishchuk N, LeLorier J, Alloul K, Makrides L, Richard J (2008). Evaluation of a workplace health program to reduce coronary risk factors. Clin Gov Int J.

[58] Milani RV, Lavie CJ (2009). Impact of worksite wellness intervention on cardiac risk factors and one-year health care costs. Am J Cardiol.

[59] Lemon SC, Zapka J, Li W, Estabrook B, Rosal M, Magner R (2010). Step ahead. Am J Prev Med.

[60] Williams AE, Stevens VJ, Albright CL, Nigg CR, Meenan RT, Vogt TM (2014). The results of a 2-year randomized trial of a worksite weight management intervention. Am J Health Promot AJHP..

[61] Lemon SC, Wang ML, Wedick NM, Estabrook B, Druker S, Schneider KL (2014). Weight gain prevention in the school worksite setting: results of a multi-level cluster randomized trial. Prev Med.

[62] Brehm BJ, Gates DM, Singler M, Succop PA, D’Alessio DA (2011). Environmental changes to control obesity: a randomized controlled trial in manufacturing companies. Am J Health Promot AJHP..

[63] French SA, Harnack LJ, Hannan PJ, Mitchell NR, Gerlach AF, Toomey TL (2010). Worksite environment intervention to prevent obesity among metropolitan transit workers. Prev Med.

[64] Goetzel RZ, Baker KM, Short ME, Pei X, Ozminkowski RJ, Wang S (2009). First-year results of an obesity prevention program at the Dow Chemical Company. J Occup Environ Med Am Coll Occup Environ Med..

[65] A Systematic Review of Internet-Based Worksite Wellness Approaches for Cardiovascular Disease Risk Management: Outcomes, Challenges & Opportunities [Internet]. [cited 2018 Sep 26]. Available from: https://journals.plos.org/plosone/article?id=10.1371/journal.pone.0083594 10.1371/journal.pone.0083594 PMC3885454 24421894

[66] Proper KI, Koning M, van der Beek AJ, Hildebrandt VH, Bosscher RJ, van Mechelen W (2003). The effectiveness of worksite physical activity programs on physical activity, physical fitness, and health. Clin J Sport Med Off J Can Acad Sport Med.

[67] Fleming P, Godwin M (2008). Lifestyle interventions in primary care. Can Fam Physician.

[68] Kolbe-Alex T (2013). E, Ev L. non-communicable disease prevention and worksite health promotion programs: a brief review. Occup Med Health Aff.

[69] Visscher TL, Bell C, Gubbels JS, Huang TT, Bryant MJ, Peeters A, et al. Challenges in lifestyle and community interventions research; a call for innovation. BMC Obes [Internet]. 2014 21 [cited 2019 Sep 15];1. Available from: https://www.ncbi.nlm.nih.gov/pmc/articles/PMC4511431/10.1186/s40608-014-0029-x PMC4511431 26217515

[70] Robroek SJ, van Lenthe FJ, van Empelen P, Burdorf A (2009). Determinants of participation in worksite health promotion programmes: a systematic review. Int J Behav Nutr Phys Act.

[71] Linnan LA, Sorensen G, Colditz G, Klar DN, Emmons KM (2001). Using theory to understand the multiple determinants of low participation in worksite health promotion programs. Health Educ Behav Off Publ Soc Public Health Educ.

[72] Seifert CM, Chapman LS, Hart JK, Perez P (2012). Enhancing intrinsic motivation in health promotion and wellness. Am J Health Promot.

[73] WHO | About 9 voluntary global targets [Internet]. WHO. [cited 2019 Sep 15]. Available from: http://www.who.int/nmh/ncd-tools/definition-targets/en/

[74] MRC-Developing and evaluating complex interventions [Internet]. [cited 2019 Nov 4]. Available from: https://mrc.ukri.org/documents/pdf/complex-interventions-guidance/

