# Supplementary material for: Effect of workplace physical activity interventions on the cardio-metabolic health of working adults: systematic review and meta-analysis
Source: Int J Behav Nutr Phys Act. 2019 Dec 19;16:134. doi: 10.1186/s12966-019-0896-0 (PMC6923867; doi:10.1186/s12966-019-0896-0)
Supplement: Supplementary file 1 — Additional file 1. Search terms. This file provides the search strategy used to obtain relevant articles from PUBMED. [file 12966_2019_896_MOESM1_ESM.docx]

**Search strategy prepared for MEDLINE**

- *Population***:** workplace OR worksite OR workers OR employees
- *Intervention*: physical* adj (activit* or exercise or fitness or wellness or training or education) OR counselling OR pedometer OR stair* OR walking OR acceleromet* OR lifestyle OR health promotion OR disease prevention OR risk reduction
- *Study design*: Randomi* controlled trial OR RCT OR cluster randomi*
