# Supplementary material for: Effect of workplace physical activity interventions on the cardio-metabolic health of working adults: systematic review and meta-analysis
Source: Int J Behav Nutr Phys Act. 2019 Dec 19;16:134. doi: 10.1186/s12966-019-0896-0 (PMC6923867; doi:10.1186/s12966-019-0896-0)
Supplement: Supplementary file 2 — Additional file 2. Study interventions. This file includes a table that describes the purpose, characteristics, interventions and results of each study included in the review. [file 12966_2019_896_MOESM2_ESM.docx]

**Table 1: Study characteristics, intervention approaches and results across various studies**

| **Study Author** | **Study purpose** | **Study Characteristics** | **Intervention Duration** | **Intervention Description** | **Control Description** | **Study Results** |
| --- | --- | --- | --- | --- | --- | --- |
| Weinhold et al | To evaluate the efficacy of a worksite lifestyle intervention on metabolic and behavioral risk  factors compared with usual care. | RCT in worksite pre-diabetic employees with a BMI more than 25; in a US university; duration- 2 years | 16 weeks | Campaigns and education: Study material for healthy diet and weight loss  Individual and behavioral change: DPP based curriculum to encourage 7% weight loss and perform at least 150 minutes of moderate to vigorous PA weekly, weekly meetings, lifestyle coaches for training in healthy eating, goal setting, group discussions and activities, booklets for diet and PA monitoring | Usual care from their health care providers and a booklet developed by the National Diabetes Education | Significant between-group difference in mean (SE) percentage weight change from baseline to postintervention (−5.5% [0.6], intervention; −0.4% [0.5], control; P < .001). In the intervention group, 32.4% met the goal of achieving 7% or more weight loss postintervention, which was significantly more than in the control group (2.9%; P = .003). |
| Siegel et al | Using a participatory process to develop an obesity intervention  appropriate for elementary school personnel. | Cluster RCT in all school employees of elementary schools of LA; duration- 2 years | 2 years | Individual and behavioral change: wellness communities for health promotions activities directed at improving diet or increasing physical activity | not described | School employees in intervention schools reduced their BMI by an average of 0.04 kg/m2, whereas employees in control schools increased their BMI by an average of 0.37 kg/m2 (intervention effect=– 0.564; P<.05). The intervention did not have a significant effect on waist–hip ratio. |
| Kim et al | To study the efficacy of a tailored text-messaging intervention for obese male participants in a  worksite weight loss program of 6 months duration. | RCT in employees from the Korean gas corporation, district heating corporation and expressway corporation with a BMI> 25kg/m2; duration- 6 months | 24 weeks | Campaigns and education: Tailored text messages for setting goals, motivating, providing nutritional tips and helpful recipes, exercise tips. Educational group sessions with printed materials on managing obesity | The comparison group received identical support as the intervention group with the exception of not receiving automatic tailored text messages. | The intervention group (n=63) had lost 1.71 kg (95% CI –2.53 to – 0.88) and the control group (n=59) had lost 1.56 kg (95% CI –2.45 to –0.66); the difference between the 2 groups was not significant (mean difference –0.15, 95% CI –1.36 to 1.07). |
| Almeida et al | To determine the effectiveness of an individually-targeted Internet-based intervention with monetary incentives (INCENT) at reducing weight of overweight and obese employees when compared to a less-intensive intervention (Livin’ My Weigh [LMW]) | Cluster RCT in employees with a BMI>=25 at worksites in Virginia; duration- 18 months | 12 months | Campaigns and education: Website with exercise videos, discussion forums, information on weight loss  Individual and behavioral change: Promotion of a healthy diet and 150 minutes of weekly physical activity through tailored emails, monetary incentive, self-monitoring logs for PA and dietary intake | Four newsletters on different exercise programs as well as a description of the eating plan and meal ideas, four group resource sessions on health, nutrition and strengthening exercises. | Participants lost an average of 2.27 lbs (p<0.001) with a BMI decrease of 0.36 kg/m2 (p<0.001) and 1.30 lbs (p<0.01) and a BMI decrease of 0.20 kg/m2 (p<0.01) in INCENT and LMW, respectively. The difference between INCENT and LMW group in weight loss and BMI reduction were not statistically significant. |
| Barham et al | To improve nutrition and physical activity of county  employees and promote weight loss. | RCT in pre-diabetic or diabetic employees at the Onondaga county; duration- 19 months | 12 weeks | Campaigns and education: reading materials for lifestyle improvement  Individual and behavioral change: weekly group sessions on diet and activity based on DPP curriculum | Same as intervention, only delayed by 3 months | The intervention group lost significant weight compared to the wait control group over the first 3 months (mean [95% CI], –2.23 kg [–3.5 to 0.97]) vs [+ 0.73 kg (+0.17 to +1.28)]. A decrease in BMI (P < .001) and waist circumference (P = .004)  No improvements in biochem markers |
| Viester et al | To evaluate the effectiveness of an individually tailored intervention for improvement in lifestyle behavior, health  indicators, and prevention and reduction of overweight among construction workers. | RCT in blue collar workers (carpenters, road workers, crane operators, and factory workers.) at a construction company in Netherlands; duration- 12 months | 6 months | Individual and behavioral change: Individual coaching sessions and personalized feedback, training instructions, self-monitoring and goal setting to support changes in activity & diet | Usual care | After 6 months, a statistically significant intervention effect was found on body weight (B ¼ 1.06, P ¼ .010), BMI (B ¼  0.32, P ¼ .010), and waist circumference (B ¼ 1.38, P ¼ .032). At 12 months, for weightrelated outcomes, these differences were slightly smaller and no longer statistically significant. |
| Limaye et al | To investigate a virtual assistance-based lifestyle intervention to reduce risk factors for Type 2 diabetes in young  employees in the information technology industry in India. | RCT in employees with ≥3 risk factors (family history of CVD, obesity, high blood pressure, impaired glucose, impaired lipids) in 2 IT industries in Pune; duration- 3 years | 1 year | Campaigns and education: Information on lifestyle modification through messages and emails, website and Facebook group | Information on lifestyle modification through mobile phone messages, three mobile phone messages and two e-mails per week. | At 6 months, the intervention group had significantly greater reductions in weight [-1.1 (95% CI -1.5, - 0.7) vs 0.5 (95% CI 0.2, 0.9) kg; P<0.001], waist circumference [-1.5 (95% CI -1.9, -1.1) vs 0.5 (95% CI 0.2, 0.7) cm; P<0.001], systolic blood pressure [-1.9 (95% CI -3.2, -0.6) vs 0.7 (95% CI -0.9, 2.3) mmHg; P=0.012], and diastolic blood pressure [-1.3 (95% CI -2.3, -0.3) vs 0.4 (95% CI -0.8, 1.5) mmHg, P=0.033] compared with the control group. Improvements were sustained at 1 year with the exception of systolic and diastolic blood pressure. The intervention group had significantly greater reductions in LDL cholesterol and a lower rise in glucose level than the control group at 1 year. |
| Morgan et al | To evaluate the feasibility and efficacy of a workplace-based weight loss program (Workplace  POWER-WP) for male shift workers. | RCT in over-weight/obese male shift workers at aluminium company; duration- 14 weeks | 3 months | Individual and behavioral change: Face-to-face intervention, website with personalized strategies for activity, weight loss handbook, pedometer, financial incentive | 14 week wait listed control group | There was a significant treatment effect for change in weight at 14- week follow-up (Pb.001; d=.34) with a mean difference between groups of 4.3 kg.  Significant intervention effects were also found for waist circumference (Pb.001, d=0.63), BMI (Pb.001, d=0.41), systolic blood pressure(P=.02, d=0.48) |
| Goetzel et al | To examine first-year results from a workplace environmental obesity prevention  program at The Dow Chemical Company. | Quasi experimental study in all employees in the manufacturing, r&d and administration departments at all sites of Dow S&T company; duration- 1 year | 1 year | Campaigns and education: dissemination of multiple health education materials (newsletters, intranet site, posters, and home mailings)  Individual and behavioral change: physical activity and weight management programs, health assessments and individual consultations, online behavior change programs, reimbursements for participation  Environmental change: point-of-choice messages like signs strategically located in front of stairwells, vending machines, and cafeterias, to encourage healthy eating and activity | No intervention | The treatment group lost 0.1 pounds while the control group gained 1.4 pounds, resulting in a net difference of 1.5 pounds between groups (p=0.0007). the average BMI score for the treatment group increased 0.1 while the control group BMI increased 0.3, thus producing a net statistically significant difference of 0.2 (p=0.0006).  There was a statistically significant net reduction in high blood pressure risk (odds ratio [OR]=2.8, p=0.00399) and actual systolic (p<0.0001) and diastolic (p=0.0004) blood pressure values for treatment subjects compared to controls. high blood glucose prevalence increased more in the treatment group compared to the control group (approaching significance with an OR=3.4, p=0.0502), and actual blood glucose levels increased significantly for treatment subjects |
| Wilson et al | To evaluate the effectiveness of FUEL Your Life, a translation of the Diabetes Prevention  Program for worksites. | Cluster RCT in locomotive maintenance employees at the railroad maintenance company; duration- 12 months | 6 months | Campaigns and education: posters and informational material  Individual and behavioral change: DPP program with health coaches  Environmental change: healthy food options in vending machines | No intervention | The FYL group did not demonstrate significant within-groups effects on either primary outcome measure (BMI .053, p ¼ .45; weight  .330, p ¼ .39), nor did it differ significantly over the course of the study from the control group for either BMI (Wald [1] ¼ 1.75, p , .17) or weight (Wald [1] ¼ 2.59, p , .11). |
| Linde et al | To implement a four-component  environmental intervention at the worksite level to positively influence weight gain among employees over a two year period. | Cluster RCT in worksite employees in six worksites in Minnesota; duration- 3 years | 2 years | Campaigns and education: monthly newsletters to promote positive behavioral messages and report news on obesity  Individual and behavioral change: Pedometer use, participation in the 10,000 steps a day program, walking challenges and clubs, Promote regular self-weighing, weighing challenges  Environmental change: changing vending food offerings, enhancing stairways, placement of scales with BMI charts, weight lockboxes, and tracking forms at various locations, healthy eating and activity information posted in multiple settings | No contact control group | The adjusted mean weight gain at intervention sites was 0.32 kg/m2, versus 0.19 kg/m 2 at control sites, for an adjusted mean increase in BMI of 0.13 kg/m2 (95% CI: -0.21, 0.46) units higher at intervention sites relative to controls; this difference was not statistically significant (p = 0.36). |
| Fernandez et al | To assess the effects of a worksite multiple-component intervention  addressing diet and physical activity on employees’ mean body mass  index (BMI) | Cluster RCT in worksite employees in nonunionized manufacturing, R&D company with multiple sites in the northeastern United States; duration- 5 years | 3 years | Campaigns and education: brochures, monthly newsletters, educational posters, websites with wellness information, awareness workshops, wellness fairs  Individual and behavioral change: orientation and gym, competitions for fitness, pedometer use  Environmental change: stickers on wending machines, healthy beverage signs, café nutrition promotion, mapping of indoor and outdoor walking routes, signs promoting stairway use & healthy eating, fitness rooms | No intervention | Within-group mean BMIs decreased by 0.54 kilograms per meter squared (P=.02) and 0.12 kilograms per meter squared (P=.73) at the intervention and control worksites, respectively, resulting in a difference in differences (DID) decrease of 0.42 kilograms per meter squared (P=.33). |
| Milani et al | To evaluate the clinical efficacy and cost-effectiveness  of a 6-month worksite health intervention | Cluster RCT in worksite employees of 2 geographically disparate work locations of a single US employer; duration- 1 year | 6 months | Campaigns and education: onsite health education, monthly newsletters and focus groups  Individual and behavioral change: lipid clinic, physician referral, membership in fitness centres, onsite classes on diet and activity, competitions and awards for milestones achieved, free health related premiums | Usual care | Significant improvements were demonstrated in body fat, diastolic blood pressure, and general health habits. There was a significant improvement in HDL cholesterol ( 13%, p 0.0001), total cholesterol/HDL cholesterol ratio ( 14%, p 0.0001). |
| Kramer et al | To determine if an evidence-based, behavioral lifestyle intervention program delivered at a worksite setting is effective in improving type 2 diabetes and CVD risk factors. | RCT in pre diabetic employees with BMI >=24 at Bayer corp in Pittsburg; duration- 18 months | 1 year | Campaigns and education: DVD and phone calls to assist in weight loss, handouts on fitness  Individual and behavioral change: DPP curriculum, face-to-face delivery sessions, self-monitoring logs, calorie counter, pedometer | 6 month wait listed control group | Those assigned to the immediate intervention had a significantly greater mean weight loss than those assigned to delayed control at 6 months (−10.4 lbs. or 5.1%, vs. −2.3 lbs. or 1%).  Those assigned to the immediate intervention had significantly greater improvements in HbA1c, systolic blood pressure, BMI and waist circumference. A significant decrease was noted in triglycerides for the immediate group but not for the control group although the between group difference was not significant. |
| Engbers et al | To present the effects of a relatively modest environmental intervention on biological cardiovascular risk factors. | Controlled trial in overweight office employees with a BMI>=23 in 2 Netherland government companies; duration- 1 year | 12 months | Campaigns and education: brochures and leaflets on healthy lifestyle  Environmental change: informational sheets placed near food products in canteen, as well as on vending machines, stairways made attractive, point of choice prompts placed at elevator doors, slim-making big mirrors on every floor | No intervention | Significant differences in change between groups (n= 540) in favor of the intervention group were found on: waist circumference, [1] total cholesterol for women (−0.35 mmol/l); [2] HDL for men at 3 months (0.05 mmol/l) and 12 months (0.10 mmol/l); and [3] the total–HDL ratio for the total intervention group at 3 and 12 months (−0.45 mmol/l). A significant difference in change in systolic BP was found in favor of the control group (∼4 mm Hg), due to an increase in the intervention group at both follow-ups. |
| Lemon et al | To test the effectiveness of a multilevel intervention on weight gain prevention among hospital employees. | Cluster RCT in employees of 6 hospitals in Massachussets; duration- 3 years | 3 years | Campaigns and education: weekly newsletter, a website and an information center with print materials and recipe books centrally located  Individual and behavioral change: periodic campaigns and challenges targeting physical activity, healthy eating and weight maintenance and loss, display and workshop series  Environmental change: signs in stairways and at elevator waiting areas, walking routes and maps created, café signs on nutritional information of foods and beverages | No intervention | There was no intervention impact on change in BMI from baseline to 12 (β=0.272; 95% CI= −0.271–0.782) or 24 months (β=0.276; 95% CI= −0.338, 0.890) in intention-to-treat analysis. |
| Chen et al | To determine the effectiveness of a pragmatic health promotion program to improve the metabolic disorders in older workers in Taiwan. | Quasi experimental study in full time older industrial workers in 3 worksites in Taiwan; duration- 24 weeks | 24 weeks | Campaigns and education: motivational lectures to improve knowledge  Individual and behavioral change: training in behavioral modifications to improve diet and increase activity, group competitions | No intervention | The intervention significantly lowered weight (intervention vs. ref = −1.22 vs. –0.30kg, p = 0.026), BMI (–0.46 vs. –0.02kg/m2, p = 0.006), and waist circ (–2.68 vs. +0.79cm, p <0.001), but had no effect on biochemical parameters. |
| Williams et al | To assess the effectiveness of a worksite management intervention for overweight and obese hotel employees. | Cluster RCT in hotel employees with a BMI>=25 in 30 hotels in Hawaii; duration- 2 years | 2 years | Individual and behavioral change: group sessions by counsellors on diet and activity  Environmental change: displays in cafeterias, break rooms, halls, stairways, and elevators, newsletters, promoting healthy food choices in café, modifying café menu, creating recipes | not described | Relative to the control condition, BMI in the intervention condition decreased by .10 per year (j&= .18) and WHtR decreased by .18 per year {p— .14) during 2 years. Results were not significant. At control hotels, employees' mean BMI was virtually unchanged during 12 months and increased by .12 during 24 months. At intervention hotels, em-ployees' mean BMI decreased by .10 during 12 months and by .28 during 24 months. |
| Moy et al | To study the  impact of a worksite health promotion programme on  serum cholesterol and dietary changes among employees  in a city in Malaysia | Quasi experimental study in security guards in a public health university and teaching hospital in KL; duration- 2 years | 2 years | Campaigns and education: pamphlets distributed to participants, group teaching sessions  Individual and behavioral change: one-to-one counselling, self-monitoring booklets, quiz programs, incentives  Environmental change: Microwave oven, water cooler, weighing scale placed in the work office | Minimal heath education through feedback of health check results through mails, distribution of standard brochures and group sessions. | The intervention group showed a statistically significant reduction in their mean total cholesterol levels as compared with the comparison group, with an intervention effect of –0.38 (95% CI = –0.63, –0.14) mmol/l. overall there was an increase in the mean LDL for both groups where this was mostly contributed by those in the lower LDLcholesterol (<4.53 mmo/l) group; participants with high levels ( 4.53 mmol/l) demonstrated reduced levels with a larger reduction in the intervention group; however, the difference  between groups was not significant (–0.26, 95% CI = –1.08, 0.56). systolic and diastolic blood pressure also improved in both groups although the difference was not statistically significant (P > 0.05) The HDL-cholesterol was reduced significantly in both groups (P < 0.05). However, the reduction was larger in the intervention. group following the reduction of total cholesterol level between follow up and baseline. There was no significant difference in the levels of triglycerides and fasting blood sugar in both groups over the 24-month study period. The BMI of the intervention group remained quite similar over the 24-month follow up; while there was a small increase in the comparison group |
| Lemon et al | To describe the effectiveness, reach and implementation of a weight gain prevention intervention among public school employees. | Cluster RCT in employees of 12 central Massachusetts public high schools; duration- 3 years | 3 years | Campaigns and education: health promotion displays and print and web-based materials  Individual and behavioral change: group walking and physical activity campaigns and challenges, walking groups, staff basketball games, healthy potluck lunches and breakfast, weight loss challenges, self-weighing programs  Environmental change: onsite fitness classes, lockers rooms, healthy lunch options, elimination and reduction of sugar-sweetened beverages in faculty lounges and point-of-purchase nutritional information in cafeterias | Print (newsletters) and electronic materials (website/email), on healthy recipes, walking maps of routes in the school building and outdoors, and educational materials on healthy eating, weight management and physical activity topics. | For both outcome variables, there were statistically significant differences at 24-month follow-up. There was a net change (difference of the difference) of −3.03 lbs. (p=.04) and of −.48 BMI units (p=.05) between intervention and comparison conditions. |
| Christensen et al | To study the impact of an intervention on weight loss among overweight health care workers. | Cluster RCT in female overweight health care workers at a Danish Municipality in Jutland; duration- 14 months | 12 months | Individual and behavioral change: advice on dietary change weight measurements, weight loss targets, strengthening exercises and initiating leisure time fitness exercise, sports  Environmental change: arrangement of fitness gym, fitness machines for abdominal and back extension, leg curls and leg press. | Monthly two-hour oral presentation during working hours. | The intervention group significantly reduced body weight by 6 kg (p < 0.001), BMI by 2.2 (p < 0.001) and body fat percentage by 2.8 (p < 0.001). Intervention group* There were no statistical reductions in the control group, resulting in significant differences between the two groups over time. Changes in blood pressure were not significant. |
| Atlantis et al | To investigate the effects of a comprehensive exercise and lifestyle intervention on physical health. | RCT in healthy but sedentary casino subjects in Australian casinos; duration- 1 year | 24 weeks | Campaigns and education: group seminars, worksite manual  Individual and behavioral change: weekly aerobic exercise, weight training prescribed to participants, one-to-one counselling, rewards, incentives | The wait-list control group was the no intervention group. | For study completers, between-group differences in the mean waist circumference (82.3 ± 9.2 versus 90.5 ± 17.8 cm, p = 0.01) remained significant, favoring the intervention. No significant effects on body mass index were found. |
| Racette et al | To evaluate the effectiveness of a worksite health promotion program on improving cardiovascular disease risk factors. | Cluster RCT in Medical Centre adult employees at worksites within a large medical center in Missouri; duration- 1 year | 1 year | Campaigns and education: seminars, newsletters, handouts  Individual and behavioral change: pedometers for participants, exercise sessions, group meetings, team competitions, rewards, incentives  Environmental change: weekly healthy snack cart, walking maps on site dietician for queries | Assessment only- personalized health reports | Additional improvements occurred at intervention worksite in BMI. Changes in BP and lipids not significant between worksite groups |
| Naito et al | To study the effect of  a workplace-based physical activity intervention program on  the blood lipid profiles of participating employees. | Controlled trial in factory employees in Japan; duration- 5 years | 5 years | Campaigns and education: posters, websites, workplace newspapers, campaign to increase activity  Individual and behavioral change: self-recorded diary, lectures on walking/exercise, sporting events, pedometers  Environmental change: construction of walking paths, distribution of maps | The company was provided only with individual intervention teaching material. | The absolute/proportional changes in HDL-cholesterol were 2.7 mg/dL (4.8%) in the intervention group and −0.6 mg/dL (−1.0%) in the control group. The differences between the two groups in the change in serum levels of HDL-cholesterol were highly significant (p < 0.001) in each analysis of covariance, in which the number of cigarettes smoked was included or excluded. |
| Prabhakaran et al | To develop and study the effects of a comprehensive CVD prevention and health promotion program on the cardiovascular health of employees. | Controlled trial in employees at Indian industrial sites; duration- 4 years | 4 years | Campaigns and education: posters and banners, handouts, booklets, and real-time videos, motivational sessions  Individual and behavioral change: dynamic group interactions, health melas (health display), special individual and group counselling sessions  Environmental change: modifications in canteen food to increase fruit intake and reduce fried food intake | All individuals with established CVD risk factors were referred to the industry-managed clinic for further follow-up. The industry management was free to organize any health promotion activities during the intervention phase. | After adjustment for all covariates, the mean difference in change between the intervention group and the control group ( intervention control) in weight was 2.8 kg (95% CI: 2.1 to 3.5 kg), and in waist circumference was 3.5 cm (95% CI: 2.7 to 4.3 cm). Similarly, the mean differences in change between the intervention group and the control group in SBP and diastolic blood pressure (DBP) were 11.8 mm Hg (95% CI: 10.1 to 13.4 mm Hg) and 8.4 mm Hg (95% CI: 7.3 to 9.4 mm Hg), respectively. The relative differences in change in mean plasma glucose and total cholesterol levels were also in favor of the intervention group, 17.4 mg/dl (95% CI:  13.6 to 21.2 mg/dl) and 11.8 mg/dl (95% CI: 7.3 to 16.3 mg/dl), respectively. The mean difference in change in HDL cholesterol was 4.1 mg/dl (95% CI: 2.9 to 6.4 mg/dl). Although mean triglyceride level decreased in the intervention group, the relative difference was not significant. |
| Jamal et al | To compare the effectiveness of a group based lifestyle modification  programme amongst obese individuals with an existing dietary counseling programme | RCT in overweight/obese employees from a local university in Melbourne; duration- 2 years | 24 weeks | Campaigns and education: seminars and group sessions  Individual and behavioral change: 6% weight loss and 150 minutes of PA based on the DPP curriculum, self-monitoring, dietary change advocacy | Hourly individual counselling every 12 weeks | No significant between group difference in weight, bmi, waist, bp, lipids, triglycerides, fasting glucose. |
| Shrivastava et al | To study the impact of the multicomponent interventions on body weight and cardiometabolic risk factors  in overweight individuals working in corporate worksites | Cluster RCT in overweight employees at 4 worksites from Delhi-NCR; duration- 6 months | 6 months | Campaigns and education: detailed sessions on healthy living, diet, PA every 15 days for about an hour, physical activity training sessions also given  Individual and behavioral change: Participants encouraged to engage in PA supported by pedometer use | Delayed intervention. Participants given general health talk twice in 6 months | Significant change in weight, BMI, waist, hip, skinfolds, triglycerides, lipids. |
| Muto et al | To evaluate the effectiveness of a health promotion program on the cardiovascular risk factors of employees | RCT in workers with at least one abnormal CVD risk factor at a building maintenance company in Japan; duration- 18 months | 1 year | Campaigns and education: Lectures, training, individual counselling, GDs, on PA, nutrition, CVD risk factors  Individual and behavioral change: Goal setting and evaluation | Annual health check-ups | Significant between group difference in weight, BMI, SBP, TC, TG. |
| Nilsson et al | To investigate the effects of a long-term comprehensive program of life-style intervention to prevent cardiovascular  disease. | RCT in nurses, cleaners, gardeners, drivers or transportation workers with a CVD risk score greater than 9 at 4 branches of Helsingborg public sector; duration- 18 months | 18 months | Campaigns and education: 16 group sessions with individual counselling by nurse, lectures, discussions, videos  Individual and behavioral change: outdoor activities for weight reduction and improved PA | Standard written and oral advice on CVD risk factors | Significant reduction trend (ANOVA) was recorded for BMI (–0.7 kg/m2 after 12 months and –0.5 kg/m2 after 18 months), diastolic blood pressure [–5.4 mm Hg (–0.09 kPa) after 12 months and –5.7 mm Hg (–0.76 kPa) after 18 months], LDL cholesterol (–0.3 mmol/l after 12 months and –0.3 mmol/l after 18 months), and the LDL:HDL ratio (–0.5 after 12 months and –0.5 after 18 months). Significant group differences for trend (ANCOVA) were recorded for BMI, diastolic blood pressure, and HDL cholesterol, all in favor of the intervention group |
| Brehm et al | To determine the effects of an environmental intervention on obesity, disease risk  factors, and dietary intake in an employee population. | Cluster RCT in employees of 8 US manufacturing companies | 1 year | Campaigns and education: Educational material and website to encourage a healthy lifestyle, binders at reception  Individual and behavioral change: health fair to introduce employees to health foods and walking paths via games, contests, magazine subscription  Environmental change: Point of decision prompts near stairs, elevators, break rooms, walking paths, changes to café menu | No intervention | No intervention effects on any outcome variables |
| Makrides et al | To determine the impact of a coronary risk factor modification program for  employees. | RCT in employees with at least 2 modifiable coronary risk factors in the Halifax area, Nova Scotia | 12 weeks | Campaigns and education: group seminars and counselling, on CVD risk factors, lifestyle, exercise and nutrition  Individual and behavioral change: exercise prescriptions, supervised exercise classes, home exercise program, monitoring participant progress, telephone follow ups. | Control participants offered the program when the study was completed | Difference in the change in BMI between the two groups was significant (p , 0:001). For total cholesterol, intervention participants the difference was not significant at six months. Intervention effects on HDL and BP were not significant. |
| French et al | To study the effects of a worksite intervention on obesity among  metropolitan transit workers | Cluster RCT in workers at 4 Minneapolis garages; 2 urban, 2 suburban; duration- 2 years | 18 months | Individual and behavioral change: daily weighing competitions, team walking competitions, fruit and vegetable intake challenges, fitness classes with individualized instructions including yoga, tai chi and personal training  Environmental change: changes to vending machine for more health food at lower costs, nutrition information displayed on healthy items, new fitness equipment, fitness room made aesthetic | No intervention | The intervention mean BMI change was −0.14 kg/m2, which was not statistically significant, compared to control garages. |
| Healy et al | To evaluate the initial and long-term impacts on cardio-metabolic health indicators of the Stand Up Victoria intervention | Cluster RCT in employees at worksites from a large public service organization in Australia; duration- 4 years | 12 months | Campaigns and education: Recruitment of a team champion, email from champion promoting intervention  Individual and behavioral change: health coach sessions, goal setting and tracking to check progress  Environmental change: sit-stand workstations | Usual practice along with assessment and feedback | Significant effects, favoring intervention, were observed at 12 months for fasting glucose (-0.34, 95%CI -0.65, -0.03, p=0.028 mmol/L) and the overall cardio-metabolic risk score (-0.16, 95%CI -0.30, -0.03, p=0.019). Other intervention effects were typically weakly in favor of the intervention group, but were non-significant and estimated with wide confidence intervals. |
