# Supplementary material for: Effect of workplace physical activity interventions on the cardio-metabolic health of working adults: systematic review and meta-analysis
Source: Int J Behav Nutr Phys Act. 2019 Dec 19;16:134. doi: 10.1186/s12966-019-0896-0 (PMC6923867; doi:10.1186/s12966-019-0896-0)
Supplement: Supplementary file 3 — Additional file 3. Exploratory Sub-group analyses. This file includes the tables describing the effects of workplace interventions on outcomes, analyzed under sub-groups. [file 12966_2019_896_MOESM3_ESM.docx]

**Table 1: Sub-group analysis of study outcomes by study designs**

| **Outcome** | **Subgroup- Study designs** | | | |
| --- | --- | --- | --- | --- |
|  | **Overall** | **RCTs only** | **cRCTs only** | **p-value for sub-group effect** |
| **Body weight** | -2.61 [-3.89, -1.33]  I2= 94%;  p-value= <0.00001 | -2.27 [-3.23, -1.31]  I2= 77%;  p-value=<0.0001 | 0.17 [-0.88, 1.23]  I2= 71%;  p-value= 0.004 | **p-value= 0.0008, I^2^= 91.1%** |
| **BMI** | -0.42 [-0.69, -0.15]  I2= 89%;  p-value= <0.00001 | -0.92 [-1.22, -0.61]  I2= 64%;  p-value= 0.007 | 0.12 [-0.12, 0.37]  I2= 71%;  p-value= 0.001 | **p-value= <0.00001,**  **I^2^= 96.2%** |
| **WC** | -1.92 [-3.25, -0.60]  I2= 92%;  p-value= <0.00001 | -1.85 [-2.26, -1.43]  I2= 0%;  p-value= 0.54 | 0.32 [-2.39, 3.04]  I2= 63%;  p-value= 0.07 | p-value= 0.12,  I^2^= 58.3% |
| **TC** | -3.75 [-9.84, 2.33]  I2= 86%;  p-value= <0.00001 | -3.04 [-7.51, 1.44]  I2= 41%;  p-value= 0.13 | 4.95 [-4.51, 14.41]  I2= 31%;  p-value= 0.23 | p-value= 0.13,  I^2^= 55.3% |
| **HDL** | 0.54 [-1.13, 2.20]  I2= 88%;  p-value= <0.00001 | 1.06 [-0.64, 2.76]  I2= 17%;  p-value= 0.31 | -1.48 [-2.61, -0.34]  I2= 0%;  p-value= 0.49 | p-value= 0.02,  I^2^= 83.1% |
| **LDL** | -3.25 [-8.00, 1.51]  I2= 75%;  p-value= <0.0001 | -2.97 [-6.08, 0.14]  I2= 0%;  p value= 0.64 | 1.80 [-2.84, 6.43]  I2= 0%;  p value= 0.78 | p-value= 0.09,  I^2^= 64.3% |
| **TG** | 0.62 [-4.82, 6.06]  I2= 55%;  p-value= 0.03 | 8.90 [-8.79, 26.59]  I2= 31%;  p-value= 0.24 | 3.46 [-5.39, 12.30]  I2= 63%;  p-value= 0.07 | p-value= 0.59,  I^2^= 0% |
| **Glucose** | -3.14 [-6.47, 0.20]  I2= 94%;  p-value= <0.00001 | -1.88 [-3.49, -0.27]  I2= 0%;  p-value= 0.56 | 0.31 [-0.60, -0.01]  I2= 0%;  p-value= 0.37 | p-value= 0.06,  I^2^= 71.9% |
| **SBP** | -2.05 [-5.59, 1.50]  I2= 93%;  p-value= <0.00001 | -1.93 [-4.25, 0.40]  I2= 47%;  p-value= 0.07 | -0.29 [-2.38, 1.81]  I2= 0%;  p-value= 0.58 | p-value= 0.30,  I^2^= 5.2% |
| **DBP** | -1.73 [-4.25, 0.79]  I2= 93%;  p-value= <0.00001 | -1.96 [-4.02, 0.11]  I2= 62%;  p-value= 0.01 | -1.05 [-2.65, 0.55]  I2= 0%;  p-value= 0.75 | p-value= 0.50,  I^2^= 0% |

**Table 2: Sub-group analysis of study outcomes by study duration**

| **Outcome** | **Subgroup- Study duration** | | | |
| --- | --- | --- | --- | --- |
|  | **Overall** | **6to12 months** | **>12 months** | **p-value for sub-group effect** |
| **Body weight** | -2.61 [-3.89, -1.33]  I2= 94%;  p-value= <0.00001 | -1.69 [-3.22, -0.16]  I2= 64%;  p-value= 0.01 | -3.08 [-4.81, -1.35]  I2= 97%;  p-value= <0.00001 | p-value= 0.24,  I^2^= 28.6% |
| **BMI** | -0.42 [-0.69, -0.15]  I2= 89%;  p-value= <0.00001 | -0.55 [-1.00, -0.09]  I2= 66%;  p-value= 0.007 | -0.37 [-0.71, -0.03]  I2= 92%;  p-value= <0.00001 | p-value= 0.54,  I^2^= 0% |
| **WC** | -1.92 [-3.25, -0.60]  I2= 92%;  p-value= <0.00001 | -1.73 [-3.53, 0.07]  I2= 79%;  p-value= 0.0003 | -2.01 [-3.83, -0.19]  I2= 95%;  p-value= <0.00001 | p-value= 0.83,  I^2^= 0% |
| **TC** | -3.75 [-9.84, 2.33]  I2= 86%;  p-value= <0.00001 | 1.76 [-5.42, 8.93]  I2= 0%;  p-value= 0.77 | -5.21 [-12.33,1.92]  I2= 89%;  p-value= <0.00001 | p-value= 0.18,  I^2^= 45.2% |
| **HDL** | 0.54 [-1.13, 2.20]  I2= 88%;  p-value= <0.00001 | -1.01 [-3.52, 1.50]  I2= 0%;  p-value= 1.00 | 0.75 [-1.04, 2.54]  I2= 90%;  p-value= <0.00001 | p-value= 0.26  I^2^= 19.6% |
| **LDL** | -3.25 [-8.00, 1.51]  I2= 75%;  p-value= <0.0001 | -6.48 [-19.07,6.10]  I2= 82%;  p-value= 0.004 | -1.73 [-4.62, 1.16]  I2= 14%;  P= 0.32 | p-value= 0.47,  I^2^= 0% |
| **TG** | 0.62 [-4.82, 6.06]  I2= 55%;  p-value= 0.03 | -5.00 [-35.66, 25.66]  - | 0.90 [-4.85, 6.65]  I2= 61%;  p-value= 0.02 | p-value= 0.71,  I^2^= 0% |
| **Glucose** | -3.14 [-6.47, 0.20]  I2= 94%;  p-value= <0.00001 | -4.00 [-14.01, 6.01]  - | -3.08 [-6.54, 0.38]  I2= 95%;  p-value= <0.00001 | p-value= 0.87,  I^2^= 0% |
| **SBP** | -2.05 [-5.59, 1.50]  I2= 93%;  p-value= <0.00001 | -1.11 [-5.41, 3.19]  I2= 76%;  p-value= 0.0008 | -2.48 [-7.08, 2.13]  I2= 94%;  p-value= <0.00001 | p-value= 0.67,  I^2^= 0% |
| **DBP** | -1.73 [-4.25, 0.79]  I2= 93%;  p-value= <0.00001 | 1.13 [-0.90, 3.16]  I2= 45%;  p-value= 0.12 | -2.77 [-5.60, 0.05]  I2=92%;  p-value= <0.00001 | p-value= 0.03,  I^2^= 79.3% |

**Table 3: Sub-group analysis of study outcomes by intervention type**

| **Outcome** | **Subgroup- Type of intervention** | | | |  |
| --- | --- | --- | --- | --- | --- |
|  | **Overall** | **At least Int A- campaign based approach** | **At least Int B- behavioral change approach** | **At least Int C- environmental change approach** | **p-value for sub-group effect** |
| **Body weight** | -2.61 [-3.89, -1.33]  I2= 94%;  p-value= <0.00001 | -2.49 [-3.85, -1.13]  I2= 95%;  p-value= <0.00001 | -3.00 [-4.54, -1.45]  I2= 95%;  p-value= <0.00001 | -6.09 [-17.03, 4.84]  I2= 97%;  p-value= <0.00001 | p-value= 0.74,  I^2^= 0% |
| **BMI** | -0.42 [-0.69, -0.15]  I2= 89%;  p-value= <0.00001 | -0.41 [-0.68, -0.14]  I2= 87%;  p-value= <0.00001 | -0.43 [-0.73, -0.12]  I2= 88%;  p-value= <0.00001 | -0.03 [-0.17, 0.11]  I2= 0%;  p-value= 0.55 | **p-value= 0.008,**  **I^2^= 79.1%** |
| **WC** | -1.92 [-3.25, -0.60]  I2= 92%;  p-value= <0.00001 | -1.97 [-3.49, -0.45]  I2= 94%;  p-value= <0.00001 | -2.15 [-3.81, -0.48]  I2= 91%;  p-value= <0.00001 | -2.52 [-7.31, 2.27]  I2= 98%;  p-value= <0.00001 | p-value= 0.97,  I^2^= 0% |
| **TC** | -3.75 [-9.84, 2.33]  I2= 86%;  p-value= <0.00001 | -4.42 [-10.78, 1.95]  I2= 87%;  p-value= <0.00001 | -3.43 [-10.84, 3.99]  I2= 87%;  p-value= <0.00001 | -16.13 [-20.14, -12.13]  I2= 8%;  p-value= <0.00001 | **p-value= 0.0007,**  **I^2^= 86.1%** |
| **HDL** | 0.54 [-1.13, 2.20]  I2= 88%;  p-value= <0.00001 | 0.54 [-1.13, 2.20]  I2= 88%;  p-value= <0.00001 | 0.54 [-1.13, 2.20]  I2= 88%;  p-value= <0.00001 | 0.80 [-1.54, 3.15]  I2= 90%;  p-value= <0.00001 | p-value= 0.98,  I^2^= 0% |
| **LDL** | -3.25 [-8.00, 1.51]  I2= 75%;  p-value= <0.0001 | -3.25 [-8.00, 1.51]  I2= 75%;  p-value= 0.0001 | -0.10 [-3.22, 3.01]  I2= 0%;  p-value= 0.69 | -7.27 [-16.14, 1.61]  I2= 77%;  p-value= 0.005 | p-value= 0.23,  I^2^= 31.3% |
| **TG** | 0.62 [-4.82, 6.06]  I2= 55%;  p-value= 0.03 | 0.62 [-4.82, 6.06]  I2= 55%;  p-value= 0.03 | 0.62 [-4.82, 6.06]  I2= 55%;  p-value= 0.03 | -3.10 [-8.67, 2.47]  I2= 54%;  p-value= 0.09 | p-value- 0.56,  I^2^= 0% |
| **Glucose** | -3.14 [-6.47, 0.20]  I2= 94%;  p-value= <0.00001 | -3.14 [-6.47, 0.20]  I2= 94%;  p-value= <0.00001 | -3.24 [-7.17, 0.70]  I2= 95%;  p-value= <0.00001 | -5.44 [-17.92, 7.04]  I2= 96%;  p-value= <0.00001 | p-value= 0.94,  I^2^= 0% |
| **SBP** | -2.05 [-5.59, 1.50]  I2= 93%;  p-value= <0.00001 | -1.78 [-5.85, 2.29]  I2= 94%;  p-value= <0.00001 | -2.70 [-6.55, 1.14]  I2= 91%;  p-value= <0.00001 | -2.08 [-10.33, 6.18]  I2= 97%;  p-value= <0.00001 | p-value= 0.95,  I^2^= 0% |
| **DBP** | -1.73 [-4.25, 0.79]  I2= 93%;  p-value= <0.00001 | -1.98 [-4.70, 0.73]  I2= 94%;  p-value= <0.00001 | -2.16 [-4.85, 0.52]  I2= 91%;  p-value= <0.00001 | -2.05 [-7.22, 3.13]  I2= 97%;  p-value= <0.00001 | p-value= 1.00,  I^2^= 0% |

**Table 4: Sub-group analysis of study outcomes by employee health status**

| **Outcome** | **Subgroup- Employees health status** | | |  |
| --- | --- | --- | --- | --- |
|  | **Overall** | **All employees** | **Target population** | **p-value for sub-group effect** |
| **Body weight** | -2.61 [-3.89, -1.33]  I2= 94%;  p-value= <0.00001 | -4.03 [-7.15, -0.90]  I2= 96%;  p-value= <0.00001 | -1.84 [-3.13, -0.55]  I2= 92%;  p-value= <0.00001 | p-value= 0.20,  I^2^= 37.9% |
| **BMI** | -0.42 [-0.69, -0.15]  I2= 89%;  p-value= <0.00001 | -0.13 [-0.45, 0.20]  I2= 79%;  p-value= <0.00001 | -0.66 [-1.07, -0.25]  I2= 92%;  p-value= <0.00001 | **p-value= 0.05,**  **I^2^= 75%** |
| **WC** | -1.92 [-3.25, -0.60]  I2= 92%;  p-value= <0.00001 | -3.3 [-6.09, -0.52]  I2= 89%;  p-value= <0.00001 | -1.06 [-2.08, -0.03]  I2= 83%;  p-value= <0.00001 | p-value= 0.14,  I^2^= 54.5% |
| **TC** | -3.75 [-9.84, 2.33]  I2= 86%;  p-value= <0.00001 | -7.91 [-15.54, -0.27]  I2= 77%;  p-value= 0.0005 | 0.65 [-5.51, 6.81]  I2= 71%;  p-value= 0.009 | p-value= 0.09,  I^2^= 65.7% |
| **HDL** | 0.54 [-1.13, 2.20]  I2= 88%;  p-value= <0.00001 | 0.53 [-1.50, 2.56]  I2= 88%;  p-value= <0.00001 | 0.41 [-1.70, 2.52]  I2= 66%;  P= 0.02 | p-value= 0.94,  I^2^= 0% |
| **LDL** | -3.25 [-8.00, 1.51]  I2= 75%;  p-value= <0.0001 | -1.93 [-6.95, 3.08]  I2= 0%;  p-value= 0.41 | -3.79 [-10.37, 2.78]  I2= 84%;  p-value= <0.00001 | p-value= 0.66,  I^2^= 0% |
| **TG** | 0.62 [-4.82, 6.06]  I2= 55%;  p-value= 0.03 | -2.16 [-8.88, 4.56]  I2= 60%;  p-value= 0.04 | 7.70 [0.16, 15.24]  I2= 0%;  p-value= 0.49 | p-value= 0.06,  I^2^= 72.7% |
| **Glucose** | -3.14 [-6.47, 0.20]  I2= 94%;  p-value= <0.00001 | -4.65 [-14.31, 5.01]  I2= 96%;  p-value= <0.00001 | -0.93 [-2.90, 1.04]  I2= 48%;  p-value= 0.10 | p-value= 0.46,  I^2^= 0% |
| **SBP** | -2.05 [-5.59, 1.50]  I2= 93%;  p-value= <0.00001 | -2.61 [-8.80, 3.58]  I2= 93%;  p-value= <0.00001 | -1.07 [-3.75, 1.61]  I2= 74%;  p-value= 0.0001 | p-value= 0.65,  I^2^= 0% |
| **DBP** | -1.73 [-4.25, 0.79]  I2= 93%;  p-value= <0.00001 | -1.49 [-5.95, 2.97]  I2= 94%;  p-value= <0.00001 | -1.64 [-3.69, 0.42]  I2= 77%;  p-value= 0.0001 | p-value= 0.95,  I^2^= 0% |
