# Supplementary material for: Effect of workplace physical activity interventions on the cardio-metabolic health of working adults: systematic review and meta-analysis
Source: Int J Behav Nutr Phys Act. 2019 Dec 19;16:134. doi: 10.1186/s12966-019-0896-0 (PMC6923867; doi:10.1186/s12966-019-0896-0)
Supplement: Supplementary file 4 — Additional file 4. Risk of bias summary for individual studies in the review. [file 12966_2019_896_MOESM4_ESM.docx]

**Risk of bias summary: review authors' judgments about each risk of bias item for each included study**

**
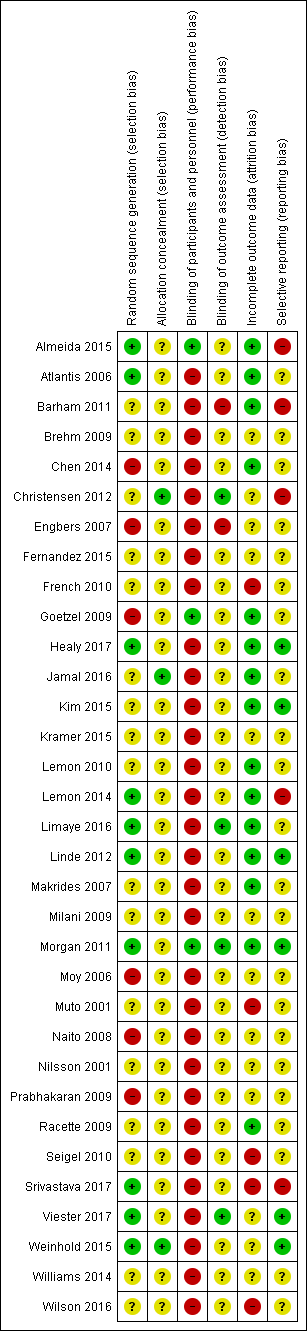
**
